# Nutritional Status in Obesity: A Comprehensive Narrative Review of Dysbiosis, Micronutrient Deficiencies and the Effects of Probiotics/Synbiotics

**DOI:** 10.3390/medicina62030458

**Published:** 2026-02-28

**Authors:** Andra-Diana Cecan, Adriana-Florinela Cătoi, Anca But, Iulia-Ioana Morar

**Affiliations:** Pathophysiology, Department of Morphofunctional Sciences, Faculty of Medicine, “Iuliu Hațieganu” University of Medicine and Pharmacy, 400012 Cluj-Napoca, Romania; andra.cecan@umfcluj.ro (A.-D.C.); anca.but@umfcluj.ro (A.B.); iulia.morar@umfcluj.ro (I.-I.M.)

**Keywords:** obesity, microbiota, micronutrient status, probiotics

## Abstract

Obesity is a chronic, relapse-prone disease often associated with comorbidities such as type 2 diabetes, dyslipidemia, and non-alcoholic fatty liver disease. Intestinal dysbiosis, defined as an imbalance in the composition and function of the gut microbiota, is commonly observed in individuals with excess body weight and plays a key role in the development of related metabolic complications. Moreover, dysbiosis can disrupt nutrient metabolism, leading to imbalances in energy homeostasis. Those affected by excess weight frequently exhibit deficiencies in essential vitamins and minerals, which further exacerbate metabolic and inflammatory dysfunctions, accelerating the progression of comorbidities. Studies have shown that the gut microbiota in individuals with obesity differs significantly from that of healthy, normal-weight individuals. Obesity often shows alterations in the relative abundance of *Firmicutes* and *Bacteroidetes* (F/B), with individual variability and reduced bacterial diversity, although the F/B ratio alone may not consistently reflect dysbiosis. Prolonged or repeated antibiotic use can further disturb the microbiota, worsening dysbiosis and contributing to the development of excess body weight by impairing energy metabolism and promoting systemic inflammation. Recent evidence suggests that probiotics are a safe and promising therapeutic approach for managing metabolic disorders. Several in vivo and clinical studies have reported a potential causal relationship between probiotic supplementation and the improvement of weight-related conditions. This narrative review aims to explore the alterations of gut microbiota in obesity and their impact on nutritional deficiencies. Additionally, it highlights the potential role of probiotics in restoring microbiota balance and improving metabolic dysfunctions related to excess body weight.

## 1. Introduction

Obesity represents a major public health problem, with a steadily increasing prevalence and a significant impact on metabolic morbidity. From a nutritional perspective, it can be regarded as a form of malnutrition driven by the chronic excessive intake of foods with low nutritional value. Beyond excess body weight, obesity is a complex metabolic disorder characterized by the progressive accumulation of energy in the form of adipose tissue, predominantly visceral, and by an increased flux of free fatty acids (FFA) into the systemic circulation.

Adipose tissue functions as an active endocrine and metabolic organ, involved in chronic low-grade inflammation and a persistent state of oxidative stress. Excess reactive oxygen species (ROS) contribute to alterations in the local and systemic inflammatory milieu by modifying the profile of inflammatory mediators, thereby promoting adipocyte hypertrophy and hyperplasia and the expansion of adipose mass.

Despite therapeutic advances, pharmacological options and surgical interventions available for body weight control are limited by variable efficacy and associated risks, which continues to make obesity management a clinical challenge. In this context, non-pharmacological interventions, particularly lifestyle modification, remain the cornerstone of current therapeutic strategies [1].

Obesity does not solely reflect an energy imbalance but is frequently associated with gut dysbiosis and micronutrient deficiencies, which may exacerbate inflammation, insulin resistance, and metabolic dysfunction. These observations have led to an increased interest in the role of the gut microbiota in host metabolic regulation and in the pathogenesis of obesity. Within this framework, modulation of the gut microbiota through nutritional interventions has emerged as a relevant research direction. Probiotics and synbiotics are considered promising strategies, with the potential to restore intestinal microbial balance, influence nutritional status, and modulate inflammatory responses and energy metabolism. We hypothesize that interventions targeting the gut microbiota may improve metabolic outcomes and nutritional status in obese patients.

Although clinical evidence is still evolving, these interventions may complement conventional lifestyle-based approaches [2].

This narrative review synthesizes current evidence from the literature to provide an integrative overview of the relationships between obesity, gut dysbiosis, micronutrient deficiencies, and the potential role of probiotics and synbiotics, highlighting insights that may inform clinical nutritional strategies and patient management.

## 2. Gut Microbiota and Gut Microbiota Dysbiosis

“Microbiota” refers to the collection of microorganisms (bacteria, archaea, microeukaryotes, and viruses) that reside in the human body and interact with the host in commensal, symbiotic, or pathological ways, playing essential roles in health and physiological balance. The “microbiome” refers to the totality of the genomes of these microorganisms. The human gut microbiota consists of seven main bacterial phyla: *Firmicutes*, *Bacteroidetes*, *Actinobacteria*, *Proteobacteria*, *Fusobacteria*, *Verrucomicrobia*, and *Cyanobacteria*, with the first three being dominant and playing a crucial role in host metabolism [3].

The microbiota is the collection of all the microorganisms in the human body. The human microbiota is distributed across various anatomical regions, including the gastrointestinal tract, skin, oral cavity, respiratory tract, urinary tract, and vaginal environment. Each of these niches harbors a distinct microbial community, finely adapted to local conditions and playing a crucial role in maintaining homeostasis and overall health. Approximately 90% of the phylogenetic categories of the healthy gut microbiota are represented by *Bacteroidetes* and *Firmicutes*, including the genera *Ruminococcus*, *Lactobacillus* and *Clostridium* species [4].

The enterotype is a classification of the gut microbiota based on the predominance of certain bacterial groups that influence the host’s metabolic and immune processes. Subsequently, changing the enterotype can have major implications for health. A multitude of factors, including aging, diet and infections, can alter a person’s enterotype. Certainly, there is a uniqueness of the microbiota, and differences in the composition and diversity of the gut microbiota vary from newborns to the elderly [4].

The composition of the microbiota is influenced by the mode of birth. In this respect, there are variations between the bacterial populations found in newborns delivered vaginally compared to those delivered by cesarean section. Vaginal birth predisposes to a bacterial composition that resembles the mother’s vaginal microbiota, being dominated by *Lactobacillus*, *Prevotella,* or *Sneathia*. In this situation, *Lactobacillus* accounts for more than 50% of the total microbiota [5]. On the other hand, those born by cesarean section acquire the bacteria present on the skin of the staff with whom they have had contact. In this situation, the microbiota is characterized by an abundance of *Staphylococcus*, *Corynebacterium* and *Propionibacterium* [5]. Compared to normal-weight individuals, obese patients exhibit reduced bacterial diversity and fecal microbial gene richness, both strongly linked to obesity and metabolic syndrome markers [6].

The gut microbiota is recognized as a microbial “active organ” or “endocrine organ”, due to its influence on the physiological functions and balances of the host organism. In this sense, the microbiota is involved in digestion, metabolic functions, protection against pathogenic microbes, synthesis of vitamins and modulation of the immune system and inflammatory reactions [4]. Anaerobic fermentation in the colon, carried out by the gut microbiota, transforms complex carbohydrates, dietary fibers, and proteins, resulting in short-chain fatty acids (SCFAs) such as acetate, propionate, and butyrate, as well as other gases like hydrogen, carbon dioxide, and methane. Dysbiosis is an alteration in the balance of the gut microbiota, characterized by a significant reduction in bacterial diversity, a proliferation of potentially pathogenic microorganisms, and a decrease in beneficial species essential for the body’s health. This imbalance affects the integrity of the intestinal barrier, facilitating the passage of bacterial compounds into the systemic circulation and initiating chronic inflammatory processes. Dysbiosis can also disrupt nutrient absorption, creating a breeding ground for the development of a wide range of conditions, including metabolic diseases and obesity [7].

## 3. Gut Microbiota Dysbiosis in Obesity

According to Sutoyo et al., some studies have reported an increased F/B ratio in individuals with obesity, which may be associated with higher SCFA concentrations. However, the relationship is not universal, and other research shows considerable variability depending on diet, population, and methodology. Therefore, the F/B ratio should be interpreted cautiously and not as a definitive marker of dysbiosis or metabolic risk [8].

Most patients with severe obesity exhibit a significant reduction in microbial diversity and gene richness, with a notable increase in bacteria from *Firmicutes*, *Proteobacteria*, *Lactobacillus*, and *Fusobacteria*, alongside a decrease in *Bacteroidetes*, *Faecalibacterium prausnitzii*, and *Akkermansia muciniphila* [9]. This shift is often accompanied by changes in the F/B ratio, although results vary among studies, and the relationship with obesity is influenced by multiple factors [10]. In grade I obesity, bacteria from the *Erysipelatoclostridiaceae* family, associated with infectious diseases, dominate, while in patients with class II obesity, *Lactobacillales*, which ferment carbohydrates into lactic acid, are more frequent. Their abundance can be enhanced by insoluble fibers, such as those found in soy [11]. Dysbiosis has been linked to obesity through multiple mechanisms. While many studies suggest associations with altered microbial composition, including *Firmicutes* and *Bacteroidetes* abundances, these patterns are not consistently observed across all populations, highlighting the complexity and individual variability of gut microbiota in obesity. Firstly, gut dysbiosis is associated with altered gut permeability, changes in bile acid metabolism, SCFAs, and metabolites affecting metabolic regulatory systems and insulin resistance [12]. Secondly, dysbiosis also reduces adenosine monophosphate-activated protein kinase (AMPK) expression in skeletal muscle and liver, favoring weight gain, aging, and excessive accumulation of ROS [13]. Thirdly, gut microbiota imbalance reduces the production of angiopoietin-like protein 4, which inhibits lipoprotein lipase, leading to excessive triglyceride deposition in the liver and muscle [14]. Finally, research on the gut microbiome has emphasized the importance of bacterial diversity in maintaining metabolic health, with alterations in microbial composition being increasingly recognized as a key feature of gut dysbiosis in obesity, and some studies appreciate proteobacterial expansion as a hallmark of an altered gut microbiome in obesity [7].

The balance of the intestinal microbiome plays a crucial role in nutrient metabolism and weight management. *Firmicutes* increase the efficiency of calorie absorption by modulating and enhancing the capacity to extract energy and the rate of metabolic degradation of energy sources, facilitating weight gain. An increased abundance of *Firmicutes* can also increase the number of lipid droplets, enhancing the absorption of fatty acids. Among the *Firmicutes* phylum, bacteria such as *Blautia hydrogenotorophica*, *Coprococcus catus*, *Eubacterium ventriosum*, *Ruminococcus bromii*, and *Ruminococcus obeum* are significantly associated with the incidence of obesity. These bacteria can degrade starch or polysaccharides and other indigestible nutritional sources, such as pectin and cellulose. Their fermentation products, SCFAs, are involved in stimulating metabolic pathways that promote energy accumulation and inflammation [8]. Furthermore, *Firmicutes* increase the levels of inflammatory molecules in the intestine, such as flagellin, lipopolysaccharides (LPS), and peptidoglycans, which accelerate the inflammatory process in patients with type 2 diabetes mellitus (T2DM). This contributes to impaired carbohydrate metabolism and the perpetuation of chronic inflammatory status, which may worsen disease progression [12].

Most butyrate-producing bacteria belong to the *Firmicutes* phylum, including species such as *Eubacterium*, *Clostridium*, *Coprococcus*, *Faecalibacterium*, *Roseburia*, and *Ruminococcus*. These bacteria are essential for the production of butyrate, a SCFA that helps maintain intestinal mucosal integrity and reduces systemic inflammation. *F. prausnitzii* and *Roseburia* species (spp.) use the enzyme butyryl-CoA: acetate-CoA transferase to synthesize butyrate, consuming acetate in the metabolic process. Butyrate synthesis predominantly occurs through the acetyl-CoA pathway in *Firmicutes*, while other phyla, such as *Bacteroidetes* and *Fusobacteria*, employ alternative pathways (e.g., lysine, succinate, glutarate) for this process. In patients with T2DM, the *Firmicutes* phylum and butyrate-producing species like *Roseburia hominis* and *F. prausnitzii* are significantly reduced, correlating with increased inflammation and decreased abundance of the *Ruminococcaceae* and *Lachnospiraceae* families. These changes are observed in both newly diagnosed and chronic diabetic patients [15].

The composition of the gut microbiota can vary across individuals, which leads to different metabolic outcomes. A common method for classifying the gut microbiome is based on enterotypes, which allow the identification of microbial composition depending on the dominance of species related to *Bacteroides* or *Prevotella*. The *Prevotella* enterotype, dominated by this genus, includes species linked in a complex trophic network. In contrast, the *Bacteroides* enterotype is divided into two types: *Bacteroides 1* (Bact1), a functional microbial composition with optimal density and high genetic diversity, dominated by *Bacteroides/Phocaeicola* and *Blautia*, and *Bacteroides* 2 (Bact2), considered dysbiotic. The latter is characterized by low microbial density and genetic diversity, high levels of *Enterobacteriaceae*, and reduced beneficial commensals, being associated with systemic inflammation, obesity, and T2DM [16]. The Bact2 enterotype represents a specific microbiome configuration characterized by an increased abundance of the *Bacteroides* genus. This microbiome profile is associated with low microbial density and correlates with more severe forms of obesity [17]. Division of the *Bacteroides* group into subgroups Bact1 and Bact2 has shown that Bact2 has reduced genetic diversity and a low number of fecal microbial cells. The prevalence of Bact2 increases with body mass index (BMI) and is associated with systemic inflammation and inflammatory diseases [17]. A study confirms that the Bact2 enterotype is linked to low microbial diversity and clinical characteristics such as severe obesity, insulin resistance, and sleep apnea. The same researchers demonstrated that initially low microbiome diversity indicates a greater potential for improvement after weight loss interventions, whether dietary or surgical [17].

The Bact2 enterotype is associated with an increased systemic inflammatory response, contributing to metabolic complications. Additionally, the presence of this enterotype is correlated with reduced functioning of the gut–brain axis, negatively influencing mental health and the predisposition to neurological disorders. Furthermore, the low levels of beneficial bacteria in this enterotype can affect the integrity of the intestinal barrier, allowing bacterial translocation and amplifying the risk of chronic inflammation in various organs and tissues [17].

Obesity-associated dysbiosis is characterized by a reduction in the abundance of genes involved in saccharolytic activity, limiting carbohydrate degradation [18]. *Prevotella* species (phylum *Bacteroidetes*) can extract calories from resistant starch and oligosaccharides, common in the diet of obese individuals, by converting them into SCFAs. These bacteria are also associated with a carbohydrate-rich diet [19]. In contrast, in non-obese individuals, the *Bacteroidetes* phylum includes bacteria such as *Bacteroides faecichinchillae*, *Bacteroides thetaiotaomicron*, *Blautia wexlerae*, *Clostridium bolteae*, and *Flavonifractor plautii*. These species are absent in obese individuals and may serve as markers for reduced risk of metabolic syndrome. SCFAs levels in fecal matter reflect the balance between their production and absorption in the colon [8].

A relevant example is *B. thetaiotaomicron*, which ferments glutamate and is inversely associated with serum glutamate levels. This suggests that an expansion of this species could contribute to reducing circulating glutamate, influencing energy metabolism, appetite, and the inflammatory response, thus impacting the development and progression of obesity [5]. Additionally, *Roseburia intestinalis*, a major butyrate producer, regulates immune responses and inflammation, maintaining an inflammatory balance that supports gut health. Butyrate stimulates the proliferation of regulatory T cells and helps maintain this inflammatory equilibrium, benefiting weight control and insulin sensitivity by inhibiting histone deacetylases and acting on free fatty acid receptors. A reduction in *Roseburia* abundance in children with hypercholesterolemia suggests a link between the microbiome and lipid metabolism, thus opening the possibility for microbiome-based interventions in obesity and dyslipidemia [20].

In patients with obesity and T2DM, muscle mass is often compromised, and sarcopenia is a commonly encountered phenomenon. The abnormal accumulation of visceral and subcutaneous adipose tissue, correlated with insulin resistance, negatively impacts muscle protein synthesis, leading to reduced capacity for muscle regeneration and maintenance. Additionally, chronic inflammation and metabolic dysfunctions associated with T2DM contribute to increased muscle catabolism. Disrupted carbohydrate metabolism, combined with a deficiency of essential nutrients, exacerbates negative protein balance, accelerating muscle loss.

The gut microbiota plays a crucial role in regulating skeletal muscle metabolism. Butyrate-producing bacteria, such as *Clostridium leptum* and *F. prausnitzii*, by reducing inflammation, can improve muscle function, while *Bifidobacterium* influences gut-muscle communication, having a positive effect on muscle mass size. Supplementation with *Bifidobacterium* may help prevent muscle loss. In contrast, species from the *Proteobacteria phylum*, which produce LPS, can increase intestinal permeability and induce systemic inflammation, negatively affecting muscle adaptation and function [21].

## 4. Dysbiosis and Inflammation

In obese individuals, recent studies have shown that the gut microbiota has an increased capacity to extract energy from food compared to lean individuals [22]. This particularity in metabolic efficiency may significantly contribute to fat accumulation and the progression of obesity, being a key factor in its development [14]. The F/B ratio may influence energy extraction from colonic fermentation in some individuals, potentially affecting SCFA production. However, this effect is not uniform, and other microbial, dietary, and host factors modulate energy balance, underscoring that F/B alone is not a reliable predictor of energy intake or obesity risk. These SCFAs not only provide energy but also play an essential role in glucose metabolism and the maintenance of energy homeostasis [8]. Interestingly, a 20% increase in *Firmicutes* and a 20% decrease in *Bacteroidetes* have been associated with an additional energy increase of 150 kcal per day [10]. The explanation is that these bacteria are capable of breaking down indigestible polysaccharides into SCFAs, which are energy providers in normal adults [14]. This increased extraction capacity can represent an evolutionary advantage during periods of famine.

Butyrate, propionate, and acetate, which constitute between 90% and 95% of the SCFAs present in the colon, act as signaling ligands between the gut microbiome and the host’s metabolism [23]. SCFAs are produced by anaerobic microorganisms through the fermentation of proteins, peptides, oligosaccharides, polysaccharides, and carbohydrates. SCFAs bind to G protein-coupled receptors (GPR41 and GPR43), with distinct affinities. GPR41 has a higher affinity for propionic, butyric, and acetic acids, while GPR43 binds all three fatty acids. Activation of GPR41 by propionic acid increases leptin expression, while binding of acetic acid to GPR43 stimulates leptin secretion in adipose tissue [8]. In obese individuals, significantly higher concentrations of acetate (in blood and feces), propionate, valerate, and butyrate (all in feces) have been observed compared to non-obese individuals. Although SCFAs have beneficial effects on health in general, in the context of obesity, elevated SCFAs concentrations may contribute to lipogenesis and lipid accumulation in adipocytes, thus facilitating the absorption and storage of energy in adipose tissue. Butyrate, in particular, seems to play a key role in the pathogenesis of obesity and T2DM, being involved in the regulation of glucose and fat metabolism [23].

SCFAs influence health through three main mechanisms: inhibiting histone deacetylase activity, signaling through GPRs that detect fatty acids, and activating anti-inflammatory mechanisms derived from the first two processes [24]. These mechanisms not only support the integrity of the intestinal barrier but also contribute to modulating the host’s immune response, thereby preventing complications associated with intestinal dysbiosis. Furthermore, SCFAs, especially butyrate, have demonstrated the ability to prevent the translocation of LPS, inflammatory molecules derived from Gram-negative bacteria, thus reducing the risk of systemic inflammation [23].

However, the beneficial effects of SCFAs may be compromised in obesity, where metabolic balance is disrupted. Paradoxically, individuals with obesity can exhibit elevated fecal SCFA levels while showing reduced efficiency of carbohydrate fermentation and SCFA signaling. Intestinal dysbiosis may alter the composition and function of gut microbiota, leading to increased SCFA production but attenuated signaling through G-protein coupled receptors (GPR41/GPR43). This impaired signaling reduces the normal metabolic effects of SCFAs, such as appetite regulation, energy expenditure, and control of lipogenesis, while still contributing to intestinal energy harvest and hepatic fat accumulation. Consequently, this disruption of gut microbiota and SCFA signaling may exacerbate insulin resistance and systemic inflammation, representing key factors in the development of metabolic syndrome [23].

Secondary intestinal dysbiosis affects the capacity for carbohydrate fermentation into SCFAs, which is essential for promoting intestinal barrier integrity and antagonizing inflammation, thus reducing lipogenesis and insulin resistance [12]. In this context, amino acids may be utilized as precursors for SCFA synthesis by bacteria. This utilization of amino acids for SCFAs production may create an imbalance in intestinal metabolism, favoring bacterial activity that transforms amino acids into SCFAs at the expense of carbohydrate fermentation, which may contribute to the progression of obesity. This situation is often observed in obese individuals with metabolic syndrome, and is linked to the high concentrations of branched-chain amino acids found in insulin-resistant states, as well as to dysregulation of incretin production, a hormone involved in regulating nutrition and energy balance [24].

## 5. Dysbiosis and Incretin Disorders

Incretin hormones, primarily represented by glucagon-like peptide 1 (GLP-1) and gastric inhibitory polypeptide (GIP), are intestinal peptides released by enteroendocrine cells. These hormones play a vital role in the control of glycemic homeostasis, with a direct impact on carbohydrate metabolism. Once released into the bloodstream, they rapidly stimulate insulin secretion from pancreatic β-cells in response to the presence of nutrients, thereby modulating postprandial glycemic variations [25]. Additionally, GLP-1 and peptide YY (PYY) attenuate intestinal motility and suppress appetite by delaying gastric emptying, which contributes to weight loss [12].

Incretin hormones are secreted following nutrient ingestion, playing a crucial role in modulating insulin secretion under hyperglycemic conditions. The GIP is predominantly synthesized and released by K cells located in the duodenum and proximal jejunum. Oral intake of carbohydrates and long-chain fatty acids activates GIP secretion, thereby facilitating an adaptive metabolic response. Moreover, the secretion of GLP-1 from intestinal L cells is stimulated by nutritional and endocrine factors, particularly following meals rich in fats and carbohydrates. Both GIP and GLP-1 exert their insulinotropic effects by binding to specific receptors on pancreatic β-cells, thereby contributing to the increase in glucose-dependent insulin secretion [26].

The incretin effect is significantly impaired in patients with T2DM, contributing to postprandial hyperglycemia [27]. Dysfunction in the secretion and action of incretins is commonly observed in the context of obesity and other metabolic disorders, particularly with hypersecretion of GIP during both fasting and after oral glucose administration [26]. In obese individuals, a diminished postprandial response to GLP-1 is noted, and this is negatively correlated with BMI [27]. Lower GLP-1 levels may promote obesity by inhibiting anorexigenic signaling, contributing to a disrupted balance in appetite and energy metabolism [26]. This decrease in GLP-1 in obesity may also be linked to insulin resistance and a reduced response of L cells to carbohydrates. These disruptions underscore the critical role of incretin hormones not only in glucose homeostasis but also in appetite regulation and energy metabolism [27].

GLP-1 secretion is reduced in both obese and T2DM patients, likely as a result of these conditions rather than as a primary cause [26]. In T2DM, pancreatic α-cell function is impaired, leading to abnormal glucagon release both during fasting and after meals [27]. However, β-cells maintain their sensitivity to GIP and GLP-1. GIP can still stimulate glucagon release even in hyperglycemic conditions, likely due to the loss of insulin’s inhibitory effect on α-cells, which is typical in T2DM [26]. Additionally, intrinsic changes in β-cells, along with elevated plasma amino acids in T2DM, may further contribute to inappropriate glucagon release. Despite these dysfunctions, GLP-1 continues to suppress glucagon secretion [27].

Furthermore, treatment with GIP receptor antagonists has been shown to reduce weight gain in rodents, while GIP receptor agonism has had similar effects through desensitization of GIP receptors on adipocytes [26]. GLP-1 receptor agonists have proven effective in reducing appetite and promoting weight loss, with some studies showing a weight loss of up to 20% [27].

**Figure 1:**
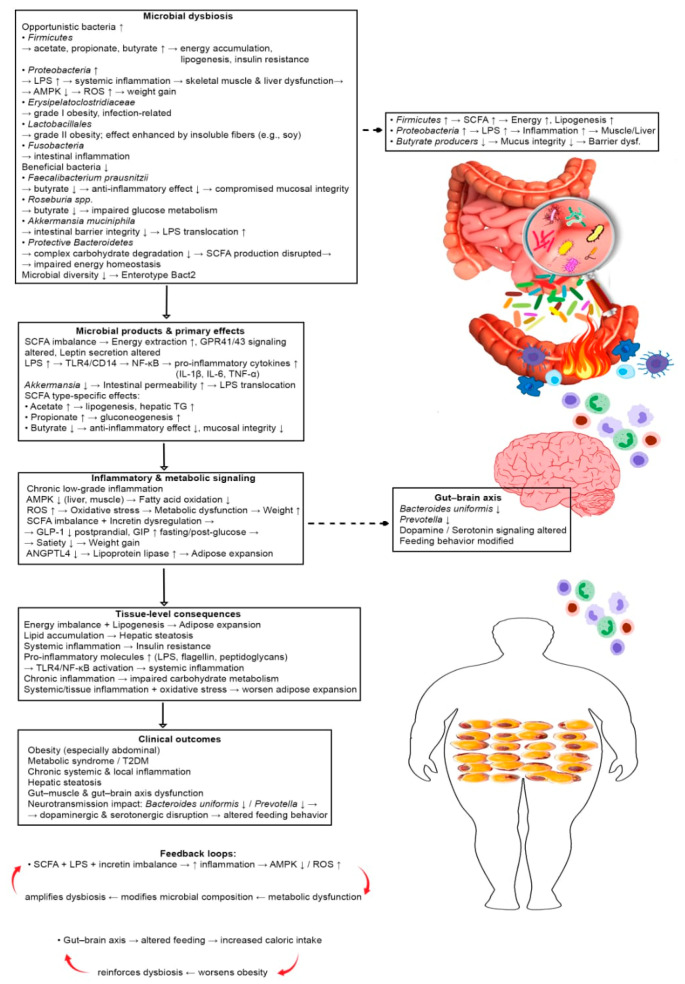
Intestinal microbiota dysbiosis and pathophysiological mechanisms in obesity.

Figure 1 illustrates the main alterations in gut microbiota associated with obesity and their mechanistic consequences on metabolism, inflammation, and tissue function. Dysbiosis is characterized by an increase in opportunistic and pathogenic bacteria, including *Firmicutes*, *Proteobacteria*, *Erysipelatoclostridiaceae*, *Lactobacillales* and *Fusobacteria*, and a decrease in beneficial bacteria such as *F. prausnitzii*, *Roseburia* spp., *A. muciniphila*, and protective *Bacteroidetes*. Reduced microbial diversity and low-density Enterotype Bact2 further destabilize the intestinal ecosystem, promoting chronic systemic inflammation. The figure highlights stepwise mechanisms. Increased fermentation by Firmicutes produces SCFAs (acetate, propionate, and butyrate), contributing to energy accumulation, lipogenesis, and insulin resistance. Excess LPS from Proteobacteria activates toll-like receptor 4 (TLR4)/CD14 signaling, triggering nuclear factor κ-light-chain-enhancer of activated B cells (NF-κB)-mediated production of pro-inflammatory cytokines (IL-1β, IL-6, tumor necrosis factor α (TNF-α)), impairing AMPK activity in muscle and liver, and enhancing triglyceride storage via reduced angiopoietin-like protein 4. Dysbiosis also affects incretin secretion (GLP-1, GIP), altering appetite regulation and glucose homeostasis. These microbiota-driven changes collectively lead to increased caloric extraction, adipose tissue expansion, hepatic steatosis, chronic systemic inflammation, and insulin resistance. Altered gut–brain and gut–muscle communication, together with disrupted neurotransmitter signaling (dopaminergic and serotonergic pathways), further influence feeding behavior and reinforce obesity.

## 6. Pro-Inflammatory Diets in Obesity and Metabolic Dysfunction

The intestinal microbiome plays a crucial role in maintaining the integrity of the mucosal barrier, and its disruption can expose the host’s immune system to bacterial products and components, resulting in a potentially harmful immune response. An increased concentration of certain intestinal bacterial species promotes the accumulation of LPS, leading to metabolic endotoxemia. This condition is accompanied by increased intestinal permeability, facilitating the translocation of bacterial products into systemic circulation.

Health-promoting bacteria in the intestine regulate the expression of tight junction proteins (such as occludin and claudin), which are essential for maintaining the integrity of the intestinal barrier. The loss of balance in the microbiome can lead to a decrease in this expression, promoting increased intestinal permeability. As a result, a persistent systemic inflammation develops, contributing to the onset of obesity [14]. Early studies in mice linked obesity with low-grade, nonspecific intestinal inflammation, activating pro-inflammatory signaling pathways like NF-κB and increasing cytokine expression, such as interleukin (IL)-1β, TNF-α, and IL-12p40. Pro-inflammatory cytokines, such as TNF-α and IL-6, are involved in inducing insulin resistance by inhibiting insulin signaling in peripheral tissues, including muscle and liver. In humans, obesity has been associated with increased systemic inflammation and changes in the innate immune system, including increased jejunal macrophages, mature dendritic cells, and natural killer cells [28]. A reduction in mucosal-associated invariant T cells leads to microbial dysbiosis and loss of intestinal integrity, promoting M1-type macrophage polarization in the intestine, which results in increased inflammation, both in mice and humans, and favors insulin resistance [29].

The consumption of a high-fat diet (HFD) stimulates the proliferation of LPS-producing bacteria, exacerbating metabolic endotoxemia and activating the inflammatory response through the CD14 receptor. Upon LPS binding, CD14, together with its co-stimulator TLR4, triggers the activation of NF-κB along the inflammatory pathway, enhancing the transcription of pro-inflammatory cytokines. These cytokines contribute to the establishment of chronic inflammation, characteristic of obesity, while persistent inflammation exacerbates metabolic dysfunctions and increases the risk of associated complications [10]. In the context of chronic inflammation, a high-fat diet also induces neutrophil migration to the intestinal mucosa, reducing the gene expression of the chemokine CXCL-1 and its receptor, CXCR-2, in the ileum. A high-fat diet not only promotes the proliferation of LPS-producing bacteria but also reduces the diversity of the intestinal microbiome, contributing to the emergence of a pro-inflammatory profile. This shift is correlated with excessive activation of the immune system and the development of insulin resistance. This reduction in neutrophil migration is associated with increased cellular proliferation and heightened DNA damage, mediated by IL-17. The chronic inflammation generated by the HFD disrupts tissue homeostasis and compromises local immune function, promoting further metabolic dysfunction [4].

Additionally, an HFD triggers the activation of neutrophils through the increased activity of elastase in these cells. In healthy individuals, metabolic endotoxemia is associated with a significant reduction of approximately 35% in systemic insulin sensitivity, and this condition can be induced by the consumption of meals rich in fats or carbohydrates such as a diet rich in ultraprocessed foods [30]. This underscores the negative impact of diet on glucose metabolism, demonstrating that the changes induced by an HFD not only affect immune responses but also negatively regulate glycemic homeostasis [10].

HFD induces dysbiosis, increasing Gram-negative LPS-producing bacteria and continuously activating TLR4, which triggers NF-κB signaling and leads to increased secretion of pro-inflammatory cytokines (IL-1β, IL-18, IL-6, IL-33, TNF-α, Interferon-γ), contributing to colonic inflammation [31]. When LPS binds to TLR4, this complex induces NF-κB activation, leading to the transcription of genes encoding pro-inflammatory cytokines. These cytokines not only cause inflammation but also interfere with insulin signaling, amplifying insulin resistance and exacerbating metabolic dysfunction. HFD also reduces tight junction proteins, impairing intestinal barrier integrity, and raises hepatic bile acid secretion. These acids, transformed into secondary bile acids by gut microbiota, influence the microbiome and intestinal inflammation. Chronic high levels of secondary bile acids are linked to colonic inflammation and barrier dysfunction [31].

A fructose-rich diet, from the consumption of soda or other foods with added sugars, can reshape the microbiota in a manner similar to a diet high in saturated fats, by increasing the proportion of *Firmicutes* and *Proteobacteria*, while significantly decreasing *Bacteroidetes* in juvenile mice. This leads to a major source of LPS from *Proteobacteria*, which contributes to low-grade inflammation through the stimulation of pro-inflammatory cytokines. High concentrations of LPS can increase intestinal permeability, affecting the intestinal barrier and leading to hepatic steatosis and inflammation [32]. The structure of LPS varies between bacterial species, influencing immunogenicity and effects on the host. For instance, Bact2 in the gut produces LPS that differs from *Escherichia coli*, a member of *Proteobacteria*, inducing different immune responses. This microbial variation may contribute differently to metabolic inflammation associated with obesity and T2DM. In particular, *Enterobacteriaceae* within *Proteobacteria*, such as *E. coli*, have stronger endotoxin properties, and an HFD can increase *Enterobacteriaceae*, supporting aerotolerant bacteria and causing intestinal inflammation. The translocation of LPS into circulation, enhanced by HFD consumption, worsens inflammation in adipose tissue and interferes with insulin and leptin actions, highlighting the link between diet and metabolic health. One study established a connection between HFD and commensal *E. coli*, which appears necessary for initiating the inflammatory response associated with this diet. In this study, increased expression of TNF-α in adipose tissue and CD68 in the liver was observed, and these changes were present only in combination with *E. coli* and HFD [33].

Secondary bile acids, such as deoxycholic acid, are produced from primary bile by the gut microbiome and can cause inflammation in the colon. These activate the inflammasome, a protein complex that contributes to chronic inflammation of the intestine and intestinal barrier dysfunction, amplifying negative effects on metabolism. Intestinal-derived metabolites, such as LPS, can directly affect extra-intestinal tissues, including adipose tissue, liver, pancreas, and skeletal muscles. Increased intestinal permeability exposes the body to microbial products, with LPS reaching the liver through the portal circulation, where it activates CD14 receptors. High-density lipoprotein (HDL), especially HDL3, paradoxically protects against LPS-mediated liver injury [31].

Circulating LPS is a biomarker for non-alcoholic fatty liver disease (NAFLD), and TLR4 activation is crucial for experimental NAFLD induced by fructose. Secondary bile acid deoxycholic acid can cause liver damage by activating the inflammasome. LPS also reaches peripheral organs like adipose tissue, influencing inflammation, matrix remodeling, and metabolic activity. In human adipose tissue, macrophages and adipocytes express TLR4/CD14 mechanisms, and LPS modulates tissue dysfunction, exacerbating inflammation and disrupting metabolic homeostasis [31].

Studies have shown that diets rich in fats and refined sugars significantly reduce insulin sensitivity, contributing to the development of metabolic syndrome. This phenomenon is correlated with increased inflammation and intestinal permeability, allowing the translocation of LPS and other microbial products into systemic circulation.

Dysbiosis, characterized by an overabundance of Gram-negative LPS-producing bacteria, activates inflammatory responses via TLR4 and promotes insulin resistance. This constant activation of the innate immune system, combined with chronic inflammation, disrupts metabolic balance and fosters the development of T2DM.

Symbiotic SCFA-producing bacteria support metabolic homeostasis and intestinal barrier integrity, while pathogenic bacteria disrupt metabolism and impair the barrier by reducing SCFA’s protective effects (including mucin secretion) and promoting a pro-inflammatory profile, contrary to the anti-inflammatory effects of symbiotic bacteria [31].

The gut–brain axis is a bidirectional network through which the gut microbiota influences central functions and behavior via neuroimmune, neuroendocrine, and metabolic mechanisms. The vagus nerve, activated by intestinal peptides like serotonin released from enteroendocrine cells and microbial metabolites such as butyrate, mediates the connection between the gastrointestinal tract and the solitary nucleus, which projects signals to brain regions involved in regulating food intake, energy homeostasis, and the dopaminergic response to reward [34]. Dysbiosis associated with obesity and other metabolic disorders is characterized by a decrease in key bacterial species, such as *Bacteroides uniformis* and *Prevotella* spp., as well as imbalances in the methionine/S-adenosylmethionine pathway, which is critical for synthesizing dopaminergic and serotonergic neurotransmitters. These changes impact neurotransmitter production, contributing to gut–brain axis dysfunctions and influencing eating behavior and neuroendocrine balance [34].

## 7. Ultra-Processed Foods, Dysbiosis, and Obesity

Ultra-processed foods (UPFs) have become increasingly prevalent in modern diets and are characterized by high energy density and low nutritional quality. This widespread consumption has significant implications for metabolic health and gut microbiota. Their consumption has been consistently associated with obesity, metabolic disturbances, and alterations in gut microbiota, highlighting the need to examine the mechanisms through which UPFs contribute to these outcomes [35,36].

UPFs contain significant amounts of added sugars, saturated fats, and sodium, while being low in fiber and protein [35]. Representative examples include sweetened beverages, ready-to-eat meals, reconstituted meat products, processed cheeses, and packaged snacks [36]. Together, these characteristics explain why frequent consumption of UPF is linked to both obesity and gut microbiota disruption.

The high energy density of these products promotes excessive caloric intake, while the combination of salt, sugar, and fat enhances palatability and stimulates overeating. In addition, the lack of fiber and high-quality proteins undermines satiety, further increasing caloric intake. Fiber deficiency reduces microbial fermentation and the production of SCFA, which are essential for maintaining intestinal health. Consequently, this deficit can compromise the intestinal barrier, alter bile acid signaling, and increase permeability, allowing proinflammatory microbes to enter the circulation [35].

Proteins in UPFs often originate from modified sources, such as hydrolyzed proteins, soy isolate, gluten, casein, and whey and may have reduced quality due to lack of essential amino acids or low digestibility [35]. In contrast, high-quality proteins are mainly found in minimally processed foods, such as eggs, salmon, lean meat, soy, and milk [37]. Consumption of UPF, through increased caloric intake, high energy density, and rapid ingestion, favors adipose tissue accumulation [35].

The NOVA classification distinguishes four groups of foods based on processing level. This framework helps to contextualize UPFs within the spectrum of food processing. Group 1 includes unprocessed or minimally processed foods, subject only to transformations necessary for safety, edibility, and palatability. Group 2 comprises processed ingredients, such as butter, oils, sugar, and salt, used to enhance the flavor of dishes. Group 3 includes processed foods obtained by combining two or three ingredients from the first two groups. Group 4 encompasses ultra-processed foods, containing five or more modified or extracted ingredients, combined with additives to enhance flavor and shelf life [37].

Rising UPF consumption is driven by the fast pace of modern life and limited time for cooking. Consequently, rapid and uncontrolled consumption can disrupt normal appetite regulation. UPFs reduce satiety through high energy density, soft texture, ingredient combinations, and low fiber content [37]. Food additives, such as emulsifiers (e.g., carboxymethylcellulose, polysorbate 80), preservatives, colorants, and artificial sweeteners, perturb the microbiota, favoring proinflammatory strains and reducing microbial diversity [36,38], thereby affecting the regulation of food intake. Sugar-fat, salt-fat, and carbohydrate-salt ratios further stimulate the desire to eat and increase caloric intake [35].

The high energy density of UPFs drives increased ad libitum caloric intake. Soft textures and liquid forms enable rapid ingestion, reducing satiety signaling, including PYY and GLP-1. Combinations of sugars, fats, salt, and additives produce highly palatable foods that override homeostatic appetite regulation. As a result, overeating is stimulated through hedonic pathways, promoting fat accumulation. After consuming highly palatable meals, resistance to satiety signals, including cholecystokinin (CCK), leptin, and insulin, can develop [35].

Low protein density leads to the consumption of larger quantities to meet daily protein requirements. The industrial food matrix, which provides sweetness without protein contribution, further disrupts satiety signals. Fiber deficiency reduces chewing time, slows gastric emptying, and diminishes satiety signaling. Fibers are fermented by the gut microbiota to produce SCFA, which are necessary for intestinal health and regulation of energy metabolism. Deficiencies in fiber and protein therefore impair control of food intake, favoring overeating and fat accumulation [35].

Rapid consumption further contributes to excess caloric intake and adipose tissue accumulation [35]. Additionally, the high refined carbohydrate content and glycemic load of UPFs increase glycemic and insulin responses [37], affecting glucose metabolism and microbiota composition [36]. UPF composition, including additives, emulsifiers, and stabilizers, creates a highly palatable environment that promotes overeating. Microbiota alterations influence bile acid metabolism and lipid absorption, generating energetic imbalances [35].

A diet rich in UPFs alters microbiota composition and diversity, favoring proinflammatory strains and reducing beneficial species. Individuals with high UPF intake exhibit increases in bacteria associated with metabolic disorders, such as *Alloprevotella*, *Negativibacillus*, *Prevotella*, and *Sutterella*. Dysbiosis increases intestinal permeability and contributes to obesity, inflammation, and metabolic disruption [36,38].

High UPF consumption is associated with reductions in SCFA-producing bacteria, including *Lachnospira* and *Roseburia*. This imbalance affects intestinal homeostasis and increases the risk of “leaky gut,” promoting obesity and systemic inflammation. Bacterial LPS enter the circulation, inducing chronic inflammation and contributing to insulin resistance and visceral fat accumulation. Microbiota imbalance further exacerbates visceral fat accumulation, metabolic dysbiosis, and obesity [36,38].

Clinical evidence indicates that ultra-processed foods rapidly affect energy balance. In a two-week randomized crossover trial, healthy adults on an ultra-processed diet ate ~500 kcal/day more, gaining 0.9 kg and 0.4 kg of fat, with higher glucose levels, while the unprocessed diet reduced weight and fat (Table 1). These findings underscore the need to explore longer-term interventions to mitigate the metabolic risks associated with ultra-processed foods [39].

Long-term dietary patterns combined with physical activity can further improve metabolic outcomes. In the one-year PREDIMED Plus trial, overweight adults on an energy-reduced Mediterranean diet with exercise lost ~4.2 kg, improved BMI, glucose, glycated hemoglobin (HbA1c), triglycerides (TG), and HDL, and showed gut microbiota shifts linked to fat loss. Controls on a non-restricted diet lost ~0.2 kg, highlighting the benefit of diet plus activity. Controls on a non-restricted Mediterranean diet lost only ~0.2 kg, showing that without caloric restriction and structured physical activity, the diet alone produces minimal changes, emphasizing the importance of combining both strategies for meaningful weight and metabolic improvements (Table 1) [40].

Alterations in gut microbiota help explain the metabolic impact of ultra-processed foods. Zheng and colleagues in 2023 found that higher UPF intake was associated with reduced alpha diversity and lower abundances of *Akkermansia* and *Faecalibacterium*. These changes correlated with elevated C-reactive protein (CRP) and increased insulin resistance, suggesting that diet-induced imbalance in gut bacteria contributes to systemic inflammation. Such findings highlight the mechanistic link between ultra-processed food consumption, gut microbiota, and metabolic dysfunction. Together, these data support the relevance of dietary quality for maintaining microbiome health and metabolic balance (Table 1) [41].

Evidence also shows that the effects of UPFs on the microbiome may differ by sex. Cuevas-Sierra and colleagues in 2021 analyzed 359 Spanish adults using a food frequency questionnaire and reported sex-specific microbial changes: women showed increased *Acidaminococcus* and *Bifidobacterium*, whereas men exhibited higher *Blautia* and *Granulicatella*. These observations indicate that dietary patterns can modulate gut microbiota composition differently in men and women. Although no direct metabolic outcomes were measured, these differences may have implications for diet-related metabolic health. This study underscores the importance of considering gut microbiota composition when evaluating UPF consumption (Table 1) [42].

Finally, large cohort studies confirm the long-term metabolic consequences of UPF. In the United Kingdom Biobank cohort from 2022 to 2023, adults aged 40 to 69 years were followed for approximately 5 years. Higher consumption of UPFs was associated with a greater risk of overall obesity, with a hazard ratio of 1.79, and abdominal obesity, with a hazard ratio of 1.30, as well as a higher likelihood of 5% or greater increases in BMI, waist circumference, and body fat percentage. Limiting UPF consumption may therefore be an important public health strategy to prevent obesity and related metabolic disorders (Table 1) [43].

Overall, the evidence underscores the role of UPFs in promoting obesity and metabolic dysfunction through multiple microbiota-related mechanisms. Addressing these pathways is critical for understanding the link between diet, gut dysbiosis, and health outcomes, and highlights the need for further focused research on UPF consumption in humans.

**Figure 2:**
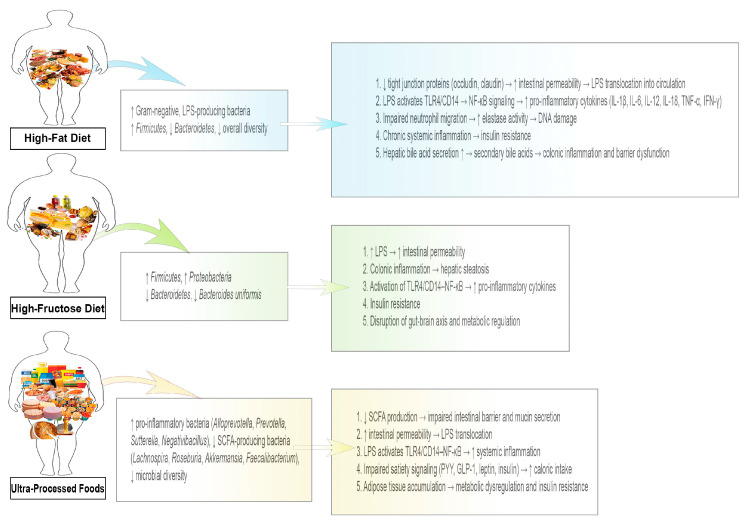
Dietary patterns, gut microbiota alterations and metabolic effects.

Figure 2 illustrates how different dietary factors influence gut microbiota composition and the resulting mechanistic effects on metabolism, intestinal barrier function, inflammation, and systemic regulation. HFD increases Gram-negative, LPS-producing bacteria (*Enterobacteriaceae*, *E. coli*), increases *Firmicutes*, decreases *Bacteroidetes,* and reduces overall microbial diversity. These changes elevate SCFA production (acetate, propionate, butyrate), promoting energy accumulation, lipogenesis, and insulin resistance. HFD also impairs tight junction proteins (occludin, claudin), increasing intestinal permeability and enabling LPS translocation. LPS activates TLR4/CD14–NF-κB signaling, increasing pro-inflammatory cytokines, impairing neutrophil migration, and enhancing elastase activity, which can damage DNA. Chronic systemic inflammation contributes to insulin resistance, while altered hepatic bile acid secretion leads to colonic inflammation and further barrier dysfunction. Fructose consumption increases *Firmicutes* and *Proteobacteria* (*Enterobacteriaceae*, *E. coli*) and decreases *Bacteroidetes*, including *Bacteroides uniformis*. Elevated LPS and SCFA imbalance impair intestinal barrier and incretin signaling. Fructose-induced dysbiosis alters GLP-1 and GIP secretion, impairing satiety, increasing appetite and disrupting glucose homeostasis. Colonic inflammation and hepatic steatosis develop and insulin resistance emerges. Gut–brain axis disruption further reinforces metabolic dysregulation. UPFs promote pro-inflammatory bacteria (*Alloprevotella*, *Prevotella*, *Sutterella*, *Negativibacillus*), reduce SCFA-producing bacteria (*Lachnospira*, *Roseburia*, *Akkermansia*, *Faecalibacterium*), and decrease microbial diversity. Reduced SCFA levels impair mucin secretion and intestinal barrier integrity, increasing intestinal permeability and LPS translocation. LPS activates TLR4/CD14–NF-κB signaling, triggering systemic inflammation. Disrupted satiety signaling (PYY, GLP-1, leptin, insulin) increases caloric intake, leading to adipose tissue accumulation, metabolic dysregulation, and insulin resistance.

## 8. Micronutrient Deficiencies and Metabolic Implications in Obesity

### 8.1. Folate

Folate and its derivatives are vital for DNA synthesis and the metabolism of homocysteine and methionine. Obese individuals often experience deficiencies in folate and vitamin B12, which can significantly affect metabolic health. The MTHFR gene, which is essential for converting folate to its active form, has been implicated in obesity, particularly with the MTHFR C677T TT genotype, which is associated with low folate levels. Furthermore, obesity enhances the activity of cytochrome P450 2E1, which uses folate as a substrate, while elevated estrogen levels may worsen folate deficiency. Folate deficiency disrupts insulin signaling and promotes insulin resistance, key mechanisms in obesity pathogenesis. Studies indicate that with each 10 kg/m^2^ increase in BMI, serum folate decreases by 15.6%, suggesting a potential benefit from folate supplementation in obese individuals, with a recommended dose of 350 µg/day [44].

In vitro research has shown that 3T3-L1 adipocytes cultured without folic acid accumulate more lipids and triglycerides, likely due to the upregulation of lipogenic genes. These findings suggest that folic acid deficiency impairs adipocyte metabolism. Elevated serum leptin levels were also observed in mice fed folic acid-deficient diets and in mature 3T3-L1 adipocytes with folic acid deficiency, further supporting the potential role of folate insufficiency in increasing obesity prevalence and contributing to chronic inflammation. Additionally, folate deficiency has been linked to energy homeostasis disruptions, which may further amplify obesity-related effects [6].

In mice fed high-fat diets, particularly those containing fructose, folic acid deficiency led to even higher serum leptin levels. Fructose is known to promote abdominal fat accumulation, and the stronger correlation between serum leptin and body weight in these experiments emphasizes fructose’s role in adipogenesis. These high-fat-fructose diets also alter lipid and carbohydrate metabolism, intensifying the consequences of folic acid deficiency. Furthermore, folate deficiency inhibits methylation and phosphatidylcholine synthesis, leading to reduced very low-density lipoprotein (VLDL) production, essential for hepatic lipid efflux. This suggests a link between folate deficiency and NAFLD, where inadequate folate status may worsen the effects of HFD on liver function [45].

### 8.2. Vitamin B

B-complex vitamins (especially B1, B3, B6, B9, and B12) play a crucial role in the development of obesity and other metabolic conditions by influencing energy metabolism, oxidative stress, inflammatory response, and lipid metabolism. Obese individuals, who often consume calorie-dense, nutrient-poor foods, exhibit significant changes in the absorption and metabolism of vitamins, including the entire B-complex group, which are essential for multiple physiological processes [46]. Deficiencies in these B vitamins are associated with a wide range of symptoms, and the mechanisms through which these deficiencies contribute to obesity are not fully elucidated.

One theory suggests that vitamin B complex (especially B1, B2, B3, B5, and B6) is essential in regulating energy metabolism, and its deficiency may disrupt this process, promoting fat accumulation and the development of obesity. Another hypothesis proposes that B vitamins (notably B2, B6, B9, and B12) have antioxidant and anti-inflammatory effects, and deficiencies in these vitamins lead to increased oxidative stress and inflammation, thereby contributing to the pathogenesis of obesity. A study showed that intestinal oxidative stress can reduce folic acid absorption, leading to its deficiency and creating a vicious cycle. Moreover, deficiencies in folic acid and vitamin B6 may elevate homocysteine levels, which inhibit lipolysis by activating the AMPK pathway. Additionally, obesity is often associated with chronic inflammation, which can alter B vitamin metabolism in various tissues [46]. Furthermore, the gut microbiota plays an essential role in the bioavailability of B vitamins through their synthesis by intestinal microbes. Although certain intestinal bacteria are capable of synthesizing vitamin B12, this microbial production does not provide a significant amount of bioavailable B12 to the human host. For example, vitamin B12 is synthesized by *Propionibacterium freudenreichii*, *Salmonella enterica*, *Listeria innocua*, as well as by lactobacilli, such as *Lactobacillus reuteri. Bifidobacterium*, particularly *Bifidobacterium bifidum* and *Bifidobacterium longum*, are renowned for synthesizing vitamin B9 in significant concentrations. The gut microbiota also contributes to the synthesis of other B vitamins, such as thiamine and riboflavin, which have a significant impact on the host’s nutritional status [47].

### 8.3. Iron

Obesity influences iron metabolism, and an elevated BMI is associated with a higher risk of iron deficiency and anemia, conditions observed in 7–52.5% of obese patients. Iron metabolism is closely linked to inflammation, with pro-inflammatory cytokines reducing erythropoiesis and promoting inflammatory anemia. In this context, IL-6 plays a significant role in Fe^2+^ sequestration through hepcidin, thus inhibiting its availability for use [48].

A key mechanism in iron imbalance in obesity is intracellular iron deficiency (IID), characterized by reduced Fe^2+^ absorption in erythroid precursors, even when iron stores appear sufficient. IID occurs due to a partial blockade of Fe^2+^ transport to the bone marrow, a process influenced by chronic inflammation associated with obesity, manifesting through elevated serum C-reactive protein. Two major mechanisms are involved in the relationship between obesity and IID: increased hepcidin synthesis in hepatocytes and adipocytes in response to pro-inflammatory cytokines, and reduced iron availability for erythroid cells, mediated by lipocalin 2 produced in mononuclear cells and adipocytes [48].

Additionally, the gut microbiota plays an important role in iron metabolism in obesity, regulating Fe^2+^ absorption by stimulating the synthesis of ferroportin (which exports Fe^2+^ from cells) and genes involved in Fe metabolism at the colon and duodenum levels. Intestinal dysbiosis, characterized by elevated levels of LPS, contributes to the chronic inflammation seen in obesity and promotes IL-6 production.

Mineral absorption can also be improved by modifying their solubility or oxidation state. For example, *L. plantarum 299v* has been shown to stimulate intestinal tissue to produce iron reductase, an enzyme that reduces plant-based iron from Fe^3+^ to Fe^2+^, a more soluble and bioavailable form [47]. Other lactobacilli have also demonstrated the ability to produce compounds with iron-reducing properties, such as p-hydroxyphenyllactic acid from *L. fermentum* [47].

Therefore, weight loss could improve iron status both by reducing adiposity and decreasing inflammation. Other lactobacilli have also demonstrated the ability to produce compounds with iron-reducing properties, such as p-hydroxyphenyllactic acid from *L. fermentum* [47].

### 8.4. Vitamin C

Vitamin C has multiple biological functions, including coenzymatic effects, and influences lipid metabolism, promoting weight-reducing processes when present in adequate amounts. Low plasma vitamin C levels are associated with a potential increase in body mass and waist circumference, as well as other negative health effects [49]. In the context of obesity, abdominal adiposity has been consistently inversely related to dietary vitamin C intake, suggesting that it may independently contribute to reducing visceral fat [50].

Moreover, vitamin C plays an essential role in fat metabolism and body composition, serving as an important cofactor in biosynthesis. Experimental animal studies have shown favorable effects on glucose metabolism and the secretion of proteins involved in obesity [50].

On the other hand, smokers and individuals with obesity or T2DM are more vulnerable to vitamin C deficiency due to increased systemic inflammation and oxidative stress, which consume vitamin C as an essential antioxidant for free radical elimination. In this context, lipid peroxidation and the accumulation of advanced lipoxidation end products are more common in obesity and T2DM, representing additional factors for vitamin C deficiency [51].

Additionally, experimental evidence suggests that vitamin C can activate the peroxisome proliferator-activated receptor (PPARs) that regulates β-oxidation of fatty acids. An animal study showed that supplementation with ascorbic acid, combined with a high-fat diet, reduced body weight, visceral adipose tissue mass, and the size of visceral adipocytes, without affecting dietary profiles [49].

Finally, studies have shown that plasma vitamin C levels are inversely correlated with BMI and waist circumference in both men and women, suggesting the need for adequate supplementation in obese patients. For example, an additional intake of 10 mg/day of vitamin C is recommended for every 10 kg increase in weight. Even with supplementation of 117 mg/day for 4 weeks, patients with high body weight (over 80 kg) do not reach adequate plasma vitamin C concentrations for optimal tissue saturation [51].

These findings emphasize the need to monitor serum vitamin C levels and adjust supplementation dosages to prevent deficiencies, especially since most obese patients do not reach optimal plasma concentrations through diet alone.

### 8.5. Vitamin A

Studies show that obese individuals have lower serum levels of retinol and carotenoids compared to those with normal weight, and dyslipidemia plays a significant role in the vitamin A deficiency associated with obesity. Research on animal models suggests that vitamin A deficiency promotes an increase in triglycerides and cholesterol; however, supplementation with vitamin A only reduces triglycerides, a phenomenon explained by insufficient antioxidant protection [52].

Vitamin A is essential for adipocyte metabolism, with approximately 15–20% of retinoids stored in adipose tissue. In the context of obesity, the expansion and differentiation of adipocytes are regulated by PPARγ, and retinoids influence this process. Vitamin A, retinaldehyde, and retinoic acid are actively involved in cell differentiation and the transcription of PPARγ. Additionally, vitamin A stimulates the production of anti-inflammatory cytokines, promoting a Th2-like response [53].

Vitamin A deficiency is commonly observed in obesity and significantly influences lipid metabolism and the inflammatory response. It can lead to abnormal increases in leptin and pro-inflammatory cytokines, promoting excessive fat deposition. Experimental studies in mice show that vitamin A deficiency increases the expression of uncoupling proteins (UCP)-1 and UCP-2 genes, which reduces thermogenesis and contributes to weight gain and elevated leptin levels. Furthermore, vitamin A deficiency stimulates adipogenesis, promoting the differentiation of preadipocytes and enhancing their survival. Thus, vitamin A deficiency encourages fat accumulation and the chronic inflammation associated with obesity, through increased leptin, resistin, and UCP expression, contributing to the low-grade inflammation characteristic of obesity [53].

Carotenoids, including beta-carotene, are important precursors of vitamin A and have anti-obesity effects by inhibiting adipogenesis and reducing the expression of PPARγ and Retinoid X Receptor (RXR) in adipose tissue. Retinoic acid, derived from beta-carotene, supports insulin sensitivity and regulates glucose metabolism through nuclear receptors of retinoic acid (RAR) and RXR, while also stimulating fatty acid oxidation and preventing lipid accumulation in adipocytes [54]. Additionally, retinoic acid influences adipocyte differentiation and regulates their survival, depending on the dose [53].

After bariatric surgery, the risk of vitamin A deficiency increases significantly in the first 30 days postoperatively due to dietary restrictions and malabsorption. This deficiency can persist in many patients even after 180 days, despite daily supplementation with 5000 IU of retinol. This ongoing deficiency negatively impacts the metabolic health of patients, contributing to long-term metabolic dysfunction [52].

Therefore, vitamin A deficiency in obesity not only promotes excessive fat deposition but also drives the associated chronic inflammation, significantly impacting metabolic balance and the overall health status of the patient [53].

### 8.6. Vitamin D

Several studies have demonstrated that the gut microbiota influences the bioavailability of fat-soluble vitamins. It contributes to the de novo synthesis of carotenoids through the mevalonate pathway and the production of proteins involved in the transport of vitamin A into enterocytes. Similarly, the intestinal absorption of vitamin D is enhanced by microbial stimulation of the vitamin D receptor expression, as well as through the modulation of fibroblast growth factor 23 activity, primarily produced by osteocytes and osteoblasts [55].

In addition to iron, vitamin D is another micronutrient that is sequestered in adipose tissue. Therefore, weight loss should improve its blood levels, especially if associated with a decrease in adiposity [47]. The high prevalence of vitamin D deficiency in obesity is thought to result from the sequestration effect of large amounts of subcutaneous fat on circulating vitamin D [56].

Vitamin D deficiency in individuals with severe obesity has been shown to be present in up to 60–80% of patients with morbid obesity before bariatric surgery procedures [56]. This high prevalence suggests a strong link between obesity and decreased vitamin D concentrations, which may influence the patient’s metabolic processes [57].

Additionally, there is an association between vitamin D levels and lipid levels. The results of a study support the idea that vitamin D plays an essential role in the adequate maintenance of apolipoprotein A-I, the main component of HDL cholesterol. In another study conducted on a healthy Belgian population, those with higher concentrations of vitamin D also had the highest plasma concentrations of apolipoprotein A-I, and 25-hydroxyvitamin D showed a positive correlation with serum HDL concentrations [56].

Recent data suggest that vitamin D deficiency may play an active role in the pathogenesis of obesity, rather than being just a consequence of it. Studies indicate that low vitamin D levels lead to increased parathyroid hormone, which stimulates lipogenesis by increasing calcium flux into adipocytes. However, more likely, the active form of vitamin D, 1.25(OH)D, inhibits adipogenesis by activating specific vitamin D receptors, which suppress adipocyte differentiation through inhibition of *C/EBP* transcription factor expression. Furthermore, 1.25(OH)D keeps the *Wnt/β*-catenin signaling pathway active, whose dysregulation promotes adipogenesis, thus limiting adipocyte formation. The high prevalence of vitamin D deficiency in obesity is well documented and appears to be due to the volumetric dilution of the vitamin in large fat deposits, serum, liver, and muscles present in individuals with obesity [58].

### 8.7. Zinc

The current literature shows that zinc influences the expression of microRNAs involved in obesity, both at the tissue and circulating levels, having an important role in regulating inflammation and metabolism. Evidence suggests a paradoxical role for zinc, as both deficiency and excess can deregulate microRNAs and promote inflammation. Further studies are needed to clarify zinc–microRNA mechanisms, to establish the optimal zinc range, and to integrate the influence of the gut microbiome, with a view to personalized therapeutic strategies in obesity [59].

At the same time, serum zinc influences carbohydrate and lipid metabolism by modulating insulin signaling and through its essential role in the synthesis, storage and secretion of insulin by pancreatic β cells. Zinc deficiency affects glucose absorption, reduces GLUT4 expression, promotes fat accumulation and contributes to insulin and leptin resistance. At the same time, zinc has an important role in regulating obesity-associated inflammation by inhibiting NF-κB and TLR4-dependent proinflammatory pathways [60].

Excessive zinc supplementation is associated with significant metabolic disturbances, favoring obesity, diabetes, hypertension, and cardiovascular disease through common mechanisms such as dyslipidemia, oxidative stress, inflammation, and insulin resistance. Although high doses of Zn (>50–150 mg/day) may be beneficial in the short term, chronic use leads to visceral fat accumulation, altered lipid and carbohydrate metabolism, and mineral imbalances. Thus, zinc has beneficial effects only within optimal dose ranges, while excessive intake becomes harmful for metabolic homeostasis and cardiovascular health [61].

### 8.8. Selenium

Selenium and selenoproteins play an important role in regulating adipose tissue function, influencing adipocyte differentiation, lipid metabolism, and redox homeostasis. Although clinical studies indicate alterations in selenoprotein levels in obese individuals, the results are often contradictory, and extrapolation of data from animal models to humans is limited [62].

Elevated selenium levels are associated with a higher risk of T2DM, an effect partially mediated by increased body mass index. Although selenium supplementation does not prevent obesity, genetically predicted selenium was associated with an increased risk of both obesity and diabetes, suggesting an indirect diabetogenic effect through influencing BMI and insulin sensitivity [63]. Another study of 15 micronutrients showed that calcium and selenium levels were associated with an increased risk of class I obesity. Within physiological limits, selenium is involved in adipogenesis and in the regulation of redox homeostasis, endoplasmic reticulum stress, inflammation, and the gut microbiome. Although some data suggest a protective role for selenium against fat accumulation, its relationship with obesity remains incompletely elucidated, and clinical evidence does not yet support the efficacy of selenium supplementation [64].

Further research, focusing on human studies and advanced human-derived models, is needed to clarify the mechanisms and clinical relevance of the relationship between selenium, selenoproteins, and obesity.

### 8.9. Magnesium

Adiposity is associated with chronic low-grade inflammation, which may exacerbate magnesium deficiency, which has been implicated as both a consequence and a determinant of metabolic dysfunction. Observational studies indicate lower serum magnesium levels in obese children and adolescents, with a consistent and strong association between hypomagnesemia, obesity, and insulin resistance. Data suggest that adiposity partially mediates this relationship and that magnesium deficiency may precede insulin resistance, being a modifiable risk factor in the pediatric population [65]. Although interventional results in adults are inconclusive, screening for magnesium in obese children and exploring nutritional interventions could have important clinical implications, requiring confirmation by longitudinal studies.

In obese subjects, a diet high in refined carbohydrates increases the dependence of carbohydrate metabolism on Mg^2+^-dependent enzymes. Mg^2+^ is essential for oxidative glucose metabolism and for thiamine activation, and its deficiency may promote energy imbalances and fat accumulation. In addition, Mg^2+^ is required for the synthesis and activation of vitamin D, and hypomagnesemia may reduce its protective effects on cardiometabolic risk [66].

Obesity, type 2 diabetes, and metabolic syndrome are closely interconnected conditions characterized by chronic inflammation, to which Mg^2+^ deficiency significantly contributes. Correcting Mg^2+^ status, including through dietary or supplemental interventions, could ameliorate this metabolic vicious cycle. However, further studies are needed to establish optimal doses and forms of Mg^2+^, as well as to clarify the molecular mechanisms and the role of the gut microbiota.

## 9. Microbiome Changes During Weight Loss

Studies on the microbiome of obese patients during weight loss reveal a “remodeling” of the gut ecosystem, marked by a relative increase in *Bacteroidetes*. While the exact taxonomic composition of a “healthy” intestinal microbiota remains uncertain, microbial diversity is critical for maintaining host homeostasis.

Diet-induced weight loss can enhance microbiota diversity and richness, as well as increase the abundance of beneficial species like *A. muciniphila*, *Lactobacillus*, and *Bifidobacterium* [17]. This shift also reduces chronic systemic inflammation, even at subclinical levels. For obese individuals on either low-carbohydrate or low-fat hypocaloric diets, a decrease in *Firmicutes* and an increase in *Bacteroidetes* have been associated with improved metabolic profiles and reduced inflammation [5,8]. Additionally, the inversion of the F/B ratio, observed after one year of diet maintenance or gastric bypass surgery, suggests a connection between microbial balance and host metabolic health [10].

Interestingly, contrasting metabolic conditions such as anorexia affect the gut microbiome differently, with reductions in *Bacteroidetes* but no significant changes in *Firmicutes*, highlighting the diverse impacts of metabolic states on gut ecosystems [10].

Regarding one study, in the intervention group, following a calorie-restricted Mediterranean diet with physical activity and behavioral support, changes in gut microbiota included an increase in the *Bacteroidetes/Firmicutes* (B/F) ratio, correlated with weight loss, high adherence to the diet, and a reduced intake of animal proteins. The genera *Butyricoccus*, *Ruminiclostridium 5*, and *Eubacterium hallii* decreased, while *Ruminococcaceae NK4A214* and *Coprobacter* increased. The genus *Coprococcus 3* was positively associated with weight, total cholesterol, and triglycerides, but negatively correlated with HDL cholesterol. Additionally, opposite changes in genera from the *Lachnospiraceae* family (*Blautia*, *Dorea*, *Roseburia*, *Coprococcus 3*) and *Ruminococcus 1* were observed between the intervention and control group [67].

In overweight and obese individuals, weight loss consistently improves gut microbiota alpha diversity and reduces intestinal permeability in a dose-dependent manner. Notable changes include increases in *Akkermansia* and *Bacteroides*, coupled with decreases in *Bifidobacterium*, without significant alterations in intestinal inflammation or SCFAs levels [68].

Although the effect of physical activity on gut microbiota remains less studied and the results are often contradictory, *Haemophilus* and *Phascolarctobacterium* show varying associations with metabolic risk factors and physical activity. These findings suggest that the observed changes in the microbiota are more influenced by the interaction between nutrient intake and energy homeostasis than by physical activity alone [67].

While formula-based diets initially cause pronounced shifts in dietary intake, most studies emphasize long-term microbiome changes observed 1–2 years after transitioning to balanced eating patterns. Weight loss promotes a microbiota profile resembling that of healthy-weight individuals, with greater α diversity and tighter gut barrier integrity. These adaptations reduce bacterial metabolites like lipopolysaccharides, enhance tight junction cohesion, limit liver exposure to inflammatory compounds, and suppress pro-inflammatory pathways [6].

However, slimming dietary interventions may induce unfavorable and sometimes transient alterations in gut microbiota. Energy restriction or high-fat/low-fiber regimens rapidly modify microbial composition and metabolic functions, without uniformly improving microbial gene richness across individuals. These shifts can reduce beneficial taxa, including *Bifidobacterium* spp. and *Faecalibacterium prausnitzii*, whose lower abundance correlates with insulin resistance and low-grade inflammation. Diet-induced microbial alterations are also linked to impaired epithelial integrity, increased gut permeability, and translocation of bacterial components such as lipopolysaccharides, promoting metabolic endotoxemia. In addition, food restriction and depleted nutritional states affect gut epithelium, gut-associated lymphoid tissue, and enteric nervous system, reflecting multidirectional feedback within the gut barrier. Thus, slimming diets can exert positive, negative, temporary, or lasting effects on gut microbiota, depending on dietary composition and host responsiveness [69,70].

## 10. Nutritional Assessment and Dietary Management in Obesity

Micronutrient deficiencies are common in obesity and contribute to metabolic dysfunctions such as insulin resistance, dyslipidemia, hepatic steatosis, anemia, and chronic inflammation. Qualitative malnutrition is characterized by excessive energy intake associated with micronutrient deficiencies, frequently observed in high-fat diets low in vitamins A, C, and folate, while chronic inflammation and excess adipose tissue impair nutrient absorption, distribution, and metabolism [71,72].

Identifying malnutrition requires assessment of dietary intake, nutritional status, and clinical, anthropometric, and biochemical parameters. Screening should focus on micronutrients with proven vulnerability in obesity, including folate, vitamins B6 and B12, iron, vitamins C, A, and D, as well as other fat-soluble vitamins in cases of malabsorption or bariatric surgery. Assessment is indicated in patients with severe obesity, metabolic comorbidities, restrictive diets, or weight loss plans, as well as before and after bariatric procedures or during pregnancy, to be conducted at the initial consultation, periodically within weight loss programs, and as part of structured follow-up [71,72].

The first step in correcting deficiencies is dietary optimization, while supplementation becomes necessary when the diet does not meet requirements, as in documented iron-deficiency anemia. Supplementation may also be indicated in patients following restrictive diets, undergoing anti-obesity pharmacotherapy, candidates for bariatric surgery, or post-operative patients, depending on the procedure type and risk of malabsorption. Early detection and timely referral to a nutrition specialist are essential for evaluating dietary intake and providing personalized recommendations tailored to each patient and the barriers to adopting a healthy lifestyle [71,72].

The most common evidence-based dietary interventions include low-fat diets (LFDs), recommending <30% of calories from fat, sometimes with caloric restriction (1200–2000 kcal/day) while allowing a higher food volume to promote satiety; low-carbohydrate diets (LCDs) providing 60–130 g of carbohydrates/day, with very-low-carbohydrate regimens (<60 g/day) replacing carbohydrates with proteins and fats; very-low-carbohydrate ketogenic diets (VLCKDs), initially limiting carbohydrates to <20 g/day and gradually increasing to 80–100 g/day, with 70–80% of calories from fat to induce ketosis; low-glycemic index diets (LGI), prioritizing whole carbohydrates to stabilize postprandial glucose; and Mediterranean diets, rich in vegetables, fruits, legumes, and whole grains, with predominantly monounsaturated fats, moderate dairy and wine intake, and limited red meat [73].

Fermented foods provide bioactive compounds and protect the gastrointestinal tract via viable microorganisms and metabolites that inhibit pathogenic overgrowth and buffer excess gastric acid and bile salts. Probiotic microorganisms produce SCFAs from prebiotic carbohydrates, which supply energy to colonic bacteria, modulate appetite hormones, and promote the growth of beneficial microbial phyla such as *Bacteroides* and *Prevotella*. SCFAs and other metabolites can activate PPARγ2, improving insulin sensitivity, lowering plasma free fatty acids and inflammation, and enhancing lipid profiles. Certain lactic acid bacteria, including *Lactobacillus plantarum*, reduce fat synthesis and storage, downregulate AMPK, FAS, ACC, and PPARγ gene expression, decrease total and LDL cholesterol and triglycerides, and increase HDL; these effects may be enhanced by concomitant intake of antioxidants or fermentation-derived metabolites. Experimental and in vitro studies show that both live probiotics and fermentation-derived metabolites regulate hepatic and adipose lipid metabolism, supporting weight reduction and metabolic health [74].

Foods rich in fiber, prebiotics, and fermented products should be encouraged across all regimens to support satiety, microbiota diversity, metabolic health, and reduced caloric intake. Soluble and insoluble fibers improve glycemic control and weight management, while prebiotics stimulate beneficial intestinal bacteria, potentially reducing inflammation and improving lipid metabolism [73].

The dietitian or nutrition specialist plays a central role in assessing and correcting deficiencies, personalizing interventions, monitoring supplementation, and supporting adherence. Screening should identify patient-specific barriers, and nutritional strategies should be gradually adapted, emphasizing fiber, fermented products, and balanced macronutrient distribution to sustain long-term metabolic health and weight management. This approach optimizes nutritional status, stabilizes metabolism, reduces complication risk, and improves long-term outcomes in obesity management [71,72].

## 11. Probiotics and Their Role in Obesity

Various studies have indicated that probiotics play a significant role in reducing obesity-related inflammation by modulating the production of inflammatory cytokines. They enhance access to amino acids, improve insulin sensitivity, stimulate pancreatic β-cells, and promote mitochondrial function through SCFA production and ROS reduction. Additionally, probiotics regulate adiposity in the liver and skeletal muscle via gut microorganisms, influencing AMPK levels [13]. Probiotics such as *Bifidobacterium* and *Lactobacillus* have beneficial effects on weight loss and glucose control. In this context, *F. prausnitzii* and *A. muciniphila* show promising results [5].

In the systematic review by L. Crovesy et al., the effects of *Lactobacillus* on body weight varied depending on the strain. Some studies reported weight or fat loss, while others found no effect or even weight gain, highlighting that the impact of *Lactobacillus* is strain-specific. Beneficial outcomes were observed with strains such as *Lactobacillus plantarum*, *L. rhamnosus*, *L. gasseri*, *L. amylovorus*, *L. acidophilus*, and *L. casei*, and with certain multi-species combinations. This strain-dependent variability supports the idea that *Lactobacillus* can modulate weight and metabolic health differently, depending on the specific strain used [75].

*Lactobacillus* strains have been increasingly studied for their potential to influence weight management and metabolic health. These bacteria can modulate the gut microbiota, improve glucose metabolism, and reduce inflammation, all of which are key factors in managing obesity and its associated conditions. Different studies suggest that the beneficial effects of *Lactobacillus* are strain-dependent [4].

Among these, *L. gasseri* BRN17 was associated with modest weight loss. Jung S.P. and colleagues reported that in a 12-week double-blind, placebo-controlled RCT involving overweight and obese adults with fasting glucose ≥ 100 mg/dL, supplementation with BRN17 slightly reduced body weight and decreased waist and hip circumferences, highlighting its modest but measurable effect (Table 2) [76].

In another investigation, researchers explored the impact of *L. delbrueckii* ssp. *bulgaricus* on metabolic parameters. In a randomized, placebo-controlled pilot trial, daily supplementation with 1 × 10^8^ CFU for 12 weeks in overweight and obese adults did not significantly alter body weight or BMI, yet it produced a notable reduction in blood triglyceride levels, suggesting a targeted benefit on lipid metabolism (Table 2) [77].

*L. sakei* represents another promising probiotic with potential anti-obesity properties, partly through inhibition of pathogenic bacterial growth. In a randomized, double-blind, placebo-controlled trial, supplementation with *L. sakei* CJLS03 at 2 × 5 × 10^9^ CFU for 12 weeks in obese and overweight adults led to a decrease in body fat mass and waist circumference, although overall body weight changes were not reported as significant (Table 2) [22].

Studies have highlighted the potential of *L. sakei* derived from Korean kimchi in mitigating obesity-related parameters. In high-fat diet-induced obese mice, an 8-week supplementation with *L. sakei* CJLS03 at three different daily doses (1 × 10^10^, 1 × 10^9^, and 1 × 10^8^ CFU) demonstrated a dose-dependent anti-obesity effect. The highest dose (1 × 10^10^ CFU/day) produced the most pronounced improvements, including reductions in body weight, fat mass, total cholesterol, and triglycerides, illustrating the strain’s capacity to positively influence metabolic biomarkers (Table 3) [78].

The combination of multiple probiotic strains has demonstrated synergistic effects in humans, enhancing weight loss, adiposity reduction, and metabolic improvements beyond single-strain interventions. In a 9-month randomized, double-blind, placebo-controlled trial, overweight adults receiving a combination of *L. acidophilus* CUL60 and CUL21, *L. plantarum* CUL66, and *Bifidobacterium animalis* subsp. *lactis* CUL34 (5 × 10^10^ CFU/day) experienced significant reductions in body weight, BMI, and waist and hip circumferences, achieved without any dietary or lifestyle modifications (Table 2) [79].

Probiotic supplementation can further modulate fat accumulation through effects on the gut microbiota. Specifically, increases in *Lachnospiraceae*, a family involved in butyrate production, have been linked to improved metabolic outcomes. Supporting this, a 12-week randomized, double-blind, placebo-controlled trial demonstrated that *L. curvatus* HY7601 and *L. plantarum* KY1032 (5 × 10^9^ CFU each) reduced body weight, visceral fat, and waist circumference in overweight and obese adults. These changes were accompanied by elevated adiponectin levels, lowered oxidized LDL, and favorable shifts in gut microbiota composition, highlighting the strains’ role in modulating host metabolism (Table 2) [80]. These strains have received regulatory recognition, with HY7601 designated as a novel food ingredient (NDI) and both strains granted GRAS status by the FDA, highlighting their safety and applicability for human consumption (Table 2) [80]. The metabolic benefits of these strains are also supported by preclinical evidence. In a 6-week experimental study in rats fed a high-fructose diet, supplementation with the same combination (*L. curvatus* HY7601 + *L. plantarum* KY1032, 5 × 10^9^ CFU each) significantly lowered plasma triglyceride levels. This effect was mediated through upregulation of apolipoprotein A-V (ApoA-V), peroxisome proliferator-activated receptor alpha (PPARα), and farnesoid X receptor (FXR), indicating enhanced lipid metabolism and demonstrating a mechanistic basis for the anti-obesity effects observed in humans (Table 3) [81].

In a single-center randomized, double-blind, placebo-controlled clinical trial, a multi-strain probiotic capsule containing *L. acidophilus*, *L. plantarum*, *B. lactis*, and *Saccharomyces boulardii* was administered for six months to individuals with type 2 diabetes. This intervention significantly reduced HbA1c levels and waist circumference compared with placebo, while improving total cholesterol and showing favorable trends in triglycerides, HDL, and LDL. Among these strains, *L. plantarum* stands out as a promising candidate for T2DM management, contributing to hepatic glucose regulation, restoration of gut microbiota balance, and reduction in low-grade inflammation, ultimately alleviating hyperglycemia and insulin resistance (Table 2) [82].

Complementing these clinical findings, preclinical studies in high-fat diet-induced obese mice demonstrated the efficacy of *L. sakei* CJLS03. Administered at 1 × 10^9^ CFU/mL for eight weeks, this strain reduced adipose tissue mass, serum cholesterol, and triglycerides, while increasing short-chain fatty acid (SCFA) levels in both serum and feces and modulating gut microbiota composition without observable toxicity. Treatment also significantly decreased body weight, fat mass, BMI, and waist circumference, and reduced the mean size of adipocytes in epididymal, subcutaneous, and mesenteric tissues. Elevated butyrate levels appeared to play a protective role against diet-induced obesity and improved insulin resistance, potentially through inhibition of lipopolysaccharide production and induction of intestinal tight junction protein expression (Table 3) [83].

Other probiotic strains have also shown consistent anti-obesity effects in diet-induced obesity murine models. Supplementation with *L. casei*, *L. fermentum*, *L. acidophilus*, *L. rhamnosus*, and *L. paracasei* decreased body weight gain, epididymal and abdominal fat, adipocyte size, and hepatic steatosis, while improving serum lipid profiles (↓ TG, ↓ total cholesterol (TC), ↓ LDL, ↑ HDL), lowering fasting glucose, decreasing leptin, and increasing adiponectin (Table 3) [84]. Similarly, *L. delbrueckii* subsp. *bulgaricus* (TCI904) reduced body weight gain and fat accumulation, improved glucose homeostasis and HOMA-IR, enhanced lipid fractions (↓ TC, ↓ TG, ↑ HDL), and attenuated anxiety-like behaviors. These findings emphasize that such effects are primarily observed in animal models, mediated through modulation of lipid and glucose metabolism, regulation of adipokines, and improvement of liver and adipose tissue function (Table 3) [85].

Furthermore, emerging evidence suggests that probiotics may influence neuroendocrine pathways linked to energy balance. For instance, *Lactobacillus reuteri* supplementation has been shown to increase circulating oxytocin levels, a hormone that regulates food intake. Impairment of oxytocin signaling has been associated with increased consumption of sweets, glucose, sucrose, sweetened beverages containing fructose, and high-fat diets, conditions frequently observed in patients with obesity [86].

*Enterobacteriaceae*, a family of bacteria associated with gastrointestinal infections, can have their adhesion to the intestinal mucosa inhibited by non-pathogenic anaerobic bacteria such as *Lactobacillus* and *Bifidobacterium*. This protective effect is largely due to the selective adhesion of *Lactobacillus* to intestinal epithelial cells, which competitively prevents colonization by pathogenic species [4].

Clinical evidence supports the metabolic benefits of *B. breve*. In a randomized, double-blind, placebo-controlled clinical trial, daily supplementation with *B. breve* B632 (DSM 24706) and BR03 (DSM 16604) at 2 × 10^9^ CFU each for 8 weeks in obese adults, children, and adolescents with HOMA-IR > 2.5 or fasting insulin > 15 mU/mL led to improved insulin sensitivity and reduced fat mass. These strains exhibit anti-inflammatory properties and stimulate immune responses against pathogens, including specific *E. coli* strains implicated in obesity. Remarkably, the beneficial effects persisted for over a month after supplementation, likely through promotion of host-protective bacterial populations and inhibition of harmful microbial groups. Short-term supplementation also improved insulin metabolism in obese children and adolescents, underscoring the potential of *B. breve* for managing obesity-related metabolic dysfunction (Table 2) [87].

Preclinical studies reinforce these findings. In high-fat diet-induced obese mice, daily supplementation with *B. breve* B-3 (10^8^–10^9^ CFU) for 8 weeks dose-dependently reduced body weight and epididymal fat accumulation. These changes were accompanied by improvements in features of metabolic syndrome, including reductions in total cholesterol, fasting glucose, insulin levels, and amelioration of NAFLD, consistent with previously reported insulin-sensitizing effects of bifidobacteria in murine models (Table 3) [88].

*F. prausnitzii* has emerged as another key gut microbe influencing metabolic health. It is closely associated with increased SCFA production, particularly butyrate, and improvements in NAFLD markers. By enhancing intestinal barrier integrity, upregulating occludin and E-cadherin, reducing permeability, and stimulating mucus production—*F. prausnitzii* contributes to gut homeostasis and metabolic regulation.

In a murine model of high-fat diet-fed NAFLD, administration of *F. prausnitzii* strains (A2-165, LB8, ZF21, PL45, and LC49) at 4 × 10^9^ CFU/mL for 12 weeks increased SCFA production, especially butyrate, improved NAFLD-related biomarkers, enhanced intestinal barrier function, and stimulated mucus production (Table 3) [89]. Across animal studies, supplementation with *F. prausnitzii* reduced hepatic steatosis, inflammation, and oxidative stress while improving glucose tolerance. Diets enriched in non-digestible carbohydrates were shown to increase *F. prausnitzii* abundance, further supporting its beneficial role in metabolic health [90].

In another murine study of high-fat diet-induced obesity, *F. prausnitzii* strains DK3, DK9, DK11, and YK1 (1 × 10^8^ CFU/150 µL) were administered for 12 weeks. Treatment reduced adipocyte size in mesenteric fat, decreased overall body fat, and improved insulin sensitivity, likely through inhibition of lipogenesis and fat accumulation. Furthermore, the supplementation modulated intestinal immunity by downregulating TLR2, TLR4, IL-1, IL-6, and MCP-1, while influencing appetite regulation via increased expression of anorexigenic genes in the hypothalamus (Table 2) [91].

**Table 2:** Human Studies on Probiotics.

Ref.	Study Type	Strain(s)	Dose (CFU/day)	Duration	Participants	Key Results
[76]	Randomized, double-blind, placebo-controlled trial	*L. gasseri* BRN17	1 × 10^10^	12 weeks	Overweight and obese adults (BMI ≥ 23 kg/m^2^, fasting glucose ≥ 100 mg/dL)	↓ body weight, ↓ waist & hip circumference but no significant differences vs. placebo
[77]	Randomized, placebo-controlled pilot trial	*L. delbrueckii* ssp. *bulgaricus*	1 × 10^8^	12 weeks	Overweight adults	↓ TG; modulation of TG in VLDL/HDL
[22]	Randomized, double-blind, placebo-controlled clinical trial	*L. sakei* CJLS03	1 × 10^10^ (2 × 5 × 10^9^)	12 weeks	Adults with obesity	↓ body fat mass, ↓ waist circumference
[79]	Randomized, parallel, double-blind, placebo-controlled trial	*L. acidophilus* CUL60 + CUL21, *L. plantarum* CUL66, *B. animalis* subsp. *lactis* CUL34	5 × 10^10^	9 months	Overweight adults	↓ body weight, ↓ waist & hip circumference
[80]	Randomized, double-blind, placebo-controlled trial	*L. curvatus* HY7601, *L. plantarum* KY1032	5 × 10^9^ each	12 weeks	Overweight adults	↓ body weight, ↓ visceral fat, ↓ waist circumference, ↑ adiponectin; ↓ LDLox; gut microbiota change
[82]	Randomized, double-blind, placebo-controlled trial	*L. acidophilus*, *L. plantarum*, *B. lactis*, *S. boulardii*	*L. acidophilus* 1.75 × 10^9^, *L. plantarum* 0.5 × 10^9^, *B. lactis* 1.75 × 10^9^, *S. boulardii* 1.5 × 10^9^	6 months	Adults with T2DM	↓ HbA1c, ↓ fasting blood glucose (FBG), ↓ TC, ↓ TG, ↑ HDL, ↓ LDL, ↓ waist circumference
[87]	Randomized, double-blind, placebo-controlled cross-over trial	*B. breve* B632, *B. breve* BR03	2 × 10^9^ each	8 weeks	Obese youths (HOMA-IR > 2.5/fasting insulin > 15 mU/mL)	↑ insulin sensitivity, ↓ fat mass, ↓ *E. coli*; improved metabolic parameters

BW = body weight; TG = triglycerides; FBG = fasting blood glucose; TC = total cholesterol; HDL = high-density lipoprotein cholesterol; LDL = low-density lipoprotein cholesterol.

**Table 3:** Animal Studies on Probiotics.

Ref.	Study Type	Strain(s)	Dose (CFU/day)	Duration	Participants	Key Results
[81]	Experimental study (rat)	*L. curvatus* HY7601 + *L. plantarum* KY1032	5 × 10^9^ each	6 weeks	Male Wistar rats, HFD	↓ TG, ↑ plasma FFA, ↑ glycerol, ↑ apoA-V, ↑ hepatic PPARα, FXR
[78]	Experimental study (murine model)	*L. sakei* CJLS03, CJB38, CJB46	1 × 10^10^/1 × 10^9^/1 × 10^8^	8 weeks	HFD mice	CJLS03 dose-dependent: ↓ body weight, ↓ visceral fat, ↓ TG, ↓ TC, ↓ aspartate aminotransferase (AST)
[83]	Experimental study (murine model)	*L. sakei* CJLS03	1 × 10^9^	8 weeks	HFD mice	↓ body weight, ↓ adipose tissue, ↑ serum/fecal SCFA; ↓ monocyte chemoattractant protein-1 (MCP-1), IL-1β, ↑ IL-10; ↓ lipogenesis genes, ↑ p-AMPK, ↓ TG & TC
[85]	Experimental study (murine model)	*L. delbrueckii* subsp. *bulgaricus* TCI904	2 × 10^7^/1 × 10^8^ CFU/kg/day	9 weeks	HFD mice	↓ body weight, ↓ fat, ↓ glucose, ↓ insulin, ↓ HOMA-IR; ↓ TC, ↓ TG, ↓ LDL, ↑ HDL; ↓ anxiety-like behavior
[84]	Experimental study (murine model)	*L. casei* NCU01105, *L. fermentum* NCU0413, *L. acidophilus* NCU433, *L. rhamnosus* NCU2217, *L. paracasei* NCU622	1 × 10^8^ CFU/mL	9 weeks	HFD mice	↓ body weight, ↓ fat, ↓ TC, ↓ TG, ↓ LDL, ↑ HDL; ↓ glucose; ↓ leptin, ↑ adiponectin; ↓ adipocyte size, ↓ hepatic steatosis
[88]	Experimental study (murine model)	*B. breve* B-3	1 × 10^8^/1 × 10^9^	8 weeks	HFD mice	Dose-dependent ↓ body weight, ↓ epididymal fat, ↑ bifidobacteria; ↑ fat metabolism genes, ↓ TC, ↓ glucose & insulin
[89]	Experimental study (murine model)	*F. prausnitzii* A2-165, LB8, ZF21, PL45, LC49	4 × 10^9^/mL	12 weeks	HFD mice with NAFLD	↑ SCFA (acetate, propionate, butyrate), improved serum lipids, ↓ hepatic steatosis & inflammation, ↑ gut barrier function
[91]	Experimental study (murine model)	*F. prausnitzii* DK3, DK9, DK11, YK1	1 × 10^8^/150 µL	12 weeks	HFD mice	↓ mesenteric adipocyte size, ↓ body fat, ↑ insulin sensitivity; ↓ TLR2, TLR4, IL-1, IL-6, MCP-1, ↑ hypothalamic anorexigenic gene expression; improved gut–brain signaling

TG = triglycerides; FFA = free fatty acids; ApoA-V = apolipoprotein A-V; PPARα = peroxisome proliferator-activated receptor alpha; FXR = farnesoid X receptor; BW = body weight; TC = total cholesterol; AST = aspartate aminotransferase; SCFA = short-chain fatty acids; MCP-1 = monocyte chemoattractant protein-1; IL-1β = interleukin-1 beta; IL-10 = interleukin-10; p-AMPK = phosphorylated AMPK; LDL = low-density lipoprotein; HDL = high-density lipoprotein.

## 12. Synbiotics and Their Role in Obesity

Synbiotics, which combine probiotics and prebiotics, act synergistically to support gut health. Probiotics provide beneficial bacteria, while prebiotics are compounds that selectively stimulate their growth. Together, they help restore gut microbiota balance, enhance metabolic processes, and potentially reduce systemic inflammation, making synbiotics a promising approach for managing conditions such as obesity and T2DM. Mechanistically, synbiotics may improve insulin function by modulating hepatic insulin signaling, reducing phosphorylation of insulin receptor substrate-1, and decreasing the production of pro-inflammatory cytokines [92].

Moreover, synbiotics can influence lipid metabolism, adipokine secretion, and energy homeostasis, providing a multifaceted approach to metabolic health.

Clinical evidence supports these metabolic benefits. In a randomized, double-blind, placebo-controlled trial, elderly patients with T2DM received a daily dose of 200 mL of a synbiotic shake containing *Lactobacillus acidophilus* and *B. bifidum* (1 × 10^8^ CFU/mL per strain) with 2 g of oligofructose for 30 days. This intervention significantly reduced fasting blood glucose and increased HDL-cholesterol compared with placebo. Additionally, synbiotic consumption led to notable decreases in serum total cholesterol and triglyceride levels, whereas the placebo group showed no comparable improvements (Table 4) [93]. These findings underscore the potential of targeted synbiotic formulations in modulating glycemic and lipid parameters in patients with established metabolic dysfunction.

The interplay between the gut microbiome and obesity is complex, influenced by multiple host and environmental factors, and many underlying mechanisms remain incompletely understood. The metabolic effects of synbiotics are highly strain-specific, reflecting differences in their capacity to modulate microbiota composition, enhance gut barrier integrity, and influence key metabolic pathways. Supporting this, a 12-week randomized, double-blind, placebo-controlled trial in children and adolescents (8–17 years) with exogenous obesity (BMI ≥ 95th percentile) examined a multi-component synbiotic sachet containing *L. acidophilus*, *L. rhamnosus*, *B. bifidum*, *B. longum*, *Enterococcus faecium*, fructo-oligosaccharides, lactulose, and a combination of vitamins. Compared with placebo, this intervention, alongside standard diet and exercise, resulted in significant improvements in anthropometric measures, including reductions in body weight, BMI, waist circumference, and waist-to-height ratio. These beneficial effects are likely mediated through modulation of gut microbiota, promoting the growth of protective bacterial populations and potentially influencing appetite regulation, energy expenditure, and adiposity, while glucose and lipid profiles remained largely unchanged over the study period (Table 4) [94]. This study highlights the feasibility of multi-strain synbiotic interventions in pediatric obesity management and suggests potential long-term benefits for metabolic programming.

In a randomized crossover trial, healthy women consumed 300 g/day of a synbiotic yogurt containing *L. acidophilus* 145, *B. longum* 913, and 1% oligofructose for three consecutive 7-week periods, alternating between control and synbiotic phases. This daily intake delivered approximately 10^6^–10^8^ CFU of *L. acidophilus* and 10^3^–10^5^ CFU of *B. longum*. After 21 weeks, participants exhibited a significant increase in HDL-cholesterol and an improved LDL/HDL ratio, while total cholesterol and LDL-cholesterol remained unchanged, demonstrating the selective benefits of the synbiotic on lipid metabolism (Table 4) [95]. These results suggest that even moderate doses of synbiotics, delivered through familiar food matrices like yogurt, can confer measurable cardiovascular benefits in healthy adults.

Similarly, in a randomized, double-blind, controlled clinical trial, healthy pregnant women in their third trimester received a synbiotic containing *L. sporogenes* (1 × 10^7^ CFU/day) combined with inulin for 9 weeks. This intervention significantly reduced serum triglycerides and VLDL-cholesterol, whereas total cholesterol, LDL, and HDL levels remained stable. In parallel, synbiotic supplementation led to a marked increase in circulating glutathione, reflecting an enhancement of systemic antioxidant status alongside the observed lipid changes (Table 4) [96]. This study emphasizes the potential of synbiotics to modulate lipid metabolism and oxidative stress during critical physiological periods such as late pregnancy.

These findings align with previous observations indicating an inverse correlation between baseline fecal *Bifidobacterium* counts and the degree of bacterial growth induced by inulin or oligofructose supplementation in healthy individuals. This suggests that prebiotic interventions may elicit stronger metabolic responses in participants with lower initial *Bifidobacterium* abundance [97]. Such personalized responses underscore the importance of baseline microbiota profiling in optimizing synbiotic therapies.

Several studies have highlighted the potential of *Lactobacillus* species, including *L. rhamnosus*, *L. casei*, and *L. plantarum*, to promote modest weight loss of approximately 5%, primarily through appetite suppression, reduced dietary intake, and attenuation of weight gain following a high-fat diet. Supporting this, a randomized, double-blind, placebo-controlled trial investigated the effects of a synbiotic containing *L. rhamnosus* CGMCC1.3724 combined with oligofructose and inulin (~3.24 × 10^8^ CFU/day via two capsules; oligofructose–inulin mixture 70:30, 300 mg/day) administered over 24 weeks, including 12 weeks of active weight loss and 12 weeks of maintenance. The intervention was well tolerated, and adherence was high throughout the study. Notably, women receiving the synbiotic exhibited significantly greater reductions in body weight and fat mass, accompanied by decreased circulating leptin levels, whereas men showed no statistically significant changes. These results indicate a sex-specific response to synbiotic supplementation in the context of obesity management (Table 4) [98]. This finding highlights the potential influence of sex hormones and differential energy metabolism on synbiotic efficacy, suggesting that tailored interventions may optimize outcomes.

*Bifidobacterium* species also play a critical role in the early prevention of obesity. Evidence suggests that children born to overweight mothers tend to have lower *Bifidobacterium* levels, and this early deficiency may be linked to an increased risk of obesity in adulthood [99]. Furthermore, increases in the abundance of *Akkermansiaceae* and *Bifidobacteriaceae* have been associated with reductions in body weight, highlighting potential mechanistic links between these bacterial families and overweight status [80].

Overall, these studies underscore the dual role of synbiotics in both prevention and management of obesity, acting through modulation of gut microbiota composition, metabolic regulation, and inflammation control.

**Table 4:** Human Studies on Synbiotics.

Ref.	Study Type	Synbiotic Composition	Dose (CFU/Day)	Duration	Participants	Key Results
[93]	Randomized, double-blind, placebo-controlled clinical trial	*L. acidophilus*, *B. bifidum*, oligofructose	1 × 10^8^ CFU/mL each strain + 2 g oligofructose	30 days	Elderly patients with type 2 diabetes and dyslipidemia	↓ FBG; ↑ HDL; ↓ TC, ↓ TG
[94]	Randomized, double-blind, placebo-controlled clinical trial	*L. acidophilus*, *L. rhamnosus*, *B. bifidum*, *B. longum*, *E. faecium*, FOS, lactulose, vitamins A, B1, B2, B6, C, E	*L. acidophilus* 4.3 × 10^8^ + *L. rhamnosus* 4.3 × 10^8^ + *B. bifidum* 4.3 × 10^8^ + *B. longum* 4.3 × 10^8^ + *E. faecium* 8.2 × 10^8^ CFU/sachet; total 2.5 × 10^9^ CFU/sachet + FOS 625 mg + lactulose 400 mg + Vitamin A (6 mg) + Vitamin B1 (1.8 mg) + Vitamin B2 (1.6 mg) + Vitamin B6 (2.4 mg) + Vitamin E (30 mg) + Vitamin C (75 mg)	12 weeks	Children and adolescents with exogenous obesity, BMI ≥ 95th percentile	↓ Body weight; ↓ BMI; ↓ waist circumference; ↓ hip circumference
[95]	Randomized cross-over trial	*L. acidophilus* 145, *B. longum* 913, oligofructose (synbiotic yogurt)	*L. acidophilus* 10^6^–10^8^ CFU + *B. longum* 10^3^–10^5^ CFU/day via 300 g/day yogurt	21 weeks (3 × 7-week periods)	Healthy women	↑ HDL
[96]	Randomized, double-blind, controlled clinical trial	*L. sporogenes*, inulin	1 × 10^7^ CFU/day + 0.04 g inulin (HPX)/g via supplement	9 weeks	Healthy pregnant women (third trimester)	↓ TG; ↓VLDL; ↑ glutathione (GSH)
[98]	Randomized, double-blind, placebo-controlled trial	*L. rhamnosus* CGMCC1.3724, oligofructose, inulin	3.24 × 10^8^ CFU/day via 2 capsules; oligofructose–inulin mixture 70:30, 300 mg/day	24 weeks (12 weeks weight loss + 12 weeks maintenance)	Obese men and women	Women: ↓ weight & fat mass, ↓ leptin; Men: no significant effect

FBG = fasting blood glucose; HDL = high-density lipoprotein cholesterol; TC = total cholesterol; TG = triglycerides; BW = body weight; BMI = body mass index; VLDL = very low-density lipoprotein; GSH = glutathione.

## 13. Conclusions

Obesity is linked to micronutrient deficiencies, chronic low-grade inflammation, and disrupted energy homeostasis, influenced by interactions between adipose tissue, liver metabolism, oxidative stress, and gut microbiota. Dysbiosis plays a key role in nutrient bioavailability and metabolic regulation. An integrated understanding of obesity, dysbiosis, and associated pathologies is essential to identify key intervention points and develop coherent, patient-tailored therapeutic strategies.

Weight loss improves microbiota diversity, intestinal barrier function, and reduces systemic inflammation. Increases in *Akkermansia*, *Bacteroides*, and *Faecalibacterium* suggest that the microbiota actively mediates metabolic responses.

Future research should clarify how microbiota and micronutrient status influence obesity, and evaluate targeted probiotics, prebiotics, and symbiotics combined with lifestyle interventions. Longitudinal clinical studies are needed to confirm their efficacy and optimize personalized treatments.

## Figures and Tables

**Table 1:** Studies on the Effects of UPFs on Body Weight, Composition and Gut Microbiota in Adults.

Ref.	Study Type	Population	Results
[39]	Randomized Controlled Trial (2-week crossover, ad libitum ultra-processed vs. unprocessed diet)	healthy adults, mean age 31 years, BMI: 27 kg/m^2^	Ultra-processed diet: increased energy intake (~500 kcal/day), increased body weight (+0.9 kg), increased fat mass (+0.4 kg), trends toward higher glucose; Unprocessed diet: decreased weight and fat mass
[40]	Randomized Clinical Trial (1-year Mediterranean diet + physical activity vs. control)	Adults 55–75 years, overweight/obese with metabolic syndrome;	Intervention (energy-reduced MedDiet + physical activity): lost ~4.2 kg Control (non-energy-restricted MedDiet): lost ~0.2 kg; reductions in BMI, fasting glucose, HbA1c, TG; ↑ HDL; microbiota changes correlated with adiposity improvements, decreases in *Butyricicoccus*, *Haemophilus*, *Ruminiclostridium*, *Eubacterium hallii*
[41]	Cross-sectional observational study	Adults (cross-sectional analyses)	↑ CRP, ↑ insulin resistance associated with dysbiosis patterns decreased alpha diversity; reduced *Akkermansia* and *Faecalibacterium* with higher UPF intake
[42]	Cross-sectional observational study	adults classified by ultra-processed food consumption via validated Food Frequency Questionnaire	high UPF consumption associated with distinct taxa differences by sex (e.g., ↑ *Acidaminococcus*, *Bifidobacterium* in women; ↑ *Blautia*, *Granulicatella* in men)
[43]	Prospective cohort study	adults aged 40–69 years (median follow-up ~5 years)	Higher UPF consumption: increased risk of overall obesity (HR ~1.79) and abdominal obesity (HR ~1.30); higher risk of ≥5% increases in BMI, waist circumference, % body fat vs. lowest UPF quartile

## Data Availability

No new data were created or analyzed in this study.

## Abbreviations

The following abbreviations are used in this manuscript:

AMPK	adenosine monophosphate-activated protein kinase
apoA-V	apolipoprotein A-V
AST	aspartate aminotransferase
B/F	Bacteroidetes/Firmicutes
Bact1	Bacteroides 1
Bact2	Bacteroides 2
BMI	body mass index
CCK	cholecystokinin
CRP	C-reactive protein
F/B	Firmicutes/Bacteroidetes
FBG	fasting blood glucose
FFA	free fatty acids
FXR	farnesoid X receptor
GIP	gastric inhibitory polypeptide
GPR	G protein-coupled receptor
GSH	glutathione
GLP-1	glucagon-like peptide 1
HDL	high-density lipoprotein
HFD	high-fat diet
HbA1c	glycated hemoglobin
IL	Interleukin
LPS	Lipopolysaccharides
MCP-1	monocyte chemoattractant protein-1
NAFLD	non-alcoholic fatty liver disease
NF-κB	nuclear factor κ-light-chain-enhancer of activated B cells
PPARα	peroxisome proliferator-activated receptor alpha
PYY	peptide YY
ROS	reactive oxygen species
SCFAs	short-chain fatty acids
spp.	species
T2DM	type 2 diabetes mellitus
TC	total cholesterol
TG	triglycerides
TLR4	Toll-like receptor 4
TNF-α	tumor necrosis factor α
UPF	ultra-processed foods
VLDL	very low-density lipoprotein

## References

[1] Emmanuel O., Duah I. (2025). Trends in Food Science & Technology Divergent roles of functional foods on anthropometric indices and gut microbiota in overweight and obese individuals: In silico approaches and multi-omics insights. Trends Food Sci. Technol..

[2] Saad M.J.A., Santos A. (2025). The Microbiota and Evolution of Obesity. Endocr. Rev..

[3] Arantes R., Bicho M., Valente A. (2020). The Possible Influence of Microbiota on Food Compulsion. J. Biomed. Sci..

[4] Konstantinidis T., Tsigalou C., Karvelas A., Stavropoulou E., Voidarou C., Bezirtzoglou E. (2020). Effects of antibiotics upon the gut microbiome: A review of the literature. Biomedicines.

[5] Vallianou N., Dalamaga M., Stratigou T., Karampela I., Tsigalou C. (2021). Do Antibiotics Cause Obesity Through Long-term Alterations in the Gut Microbiome? A Review of Current Evidence. Curr. Obes. Rep..

[6] Green M., Arora K., Prakash S. (2020). Microbial medicine: Prebiotic and probiotic functional foods to target obesity and metabolic syndrome. Int. J. Mol. Sci..

[7] Xue L., Deng Z., Luo W., He X., Chen Y. (2022). Effect of Fecal Microbiota Transplantation on Non-Alcoholic Fatty Liver Disease: A Randomized Clinical Trial. Front. Cell. Infect. Microbiol..

[8] Sutoyo D.A., Atmaka D.R., Sidabutar L.M.G.B. (2020). Dietary Factors Affecting Firmicutes and Bacteroidetes Ratio in Solving Obesity Problem: A Literature Review. Media Gizi Indones.

[9] Hanssen H.M., Fjellstad M.S., Skjevling L., Johnsen P.H., Kulseng B., Goll R., Almå K.H., Valle P.C. (2023). Randomised, placebo-controlled, double-blinded trial of fecal microbiota transplantation in severe obesity: A study protocol. BMJ Open.

[10] Tseng C.H., Wu C.Y. (2019). The gut microbiome in obesity. J. Formos. Med. Assoc..

[11] Hu J., Guo P., Mao R., Ren Z., Wen J., Yang Q., Yu J., Zhang T., Liu Y., Yan T. (2022). Gut Microbiota Signature of Obese Adults Across Different Classifications. Diabetes Metab. Syndr. Obes. Targets Ther..

[12] Kassaian N., Feizi A., Rostami S., Aminorroaya A., Yaran M., Amini M. (2020). The effects of 6 months of supplementation with probiotics and synbiotics on gut microbiota in the adults with prediabetes: A double blind randomized clinical trial. Nutrition.

[13] Jamshidi S., Masoumi S.J., Abiri B., Vafa M. (2022). The effects of synbiotic and/or vitamin D supplementation on gut-muscle axis in overweight and obese women: A study protocol for a double-blind, randomized, placebo-controlled trial. Trials.

[14] Leong K.S.W., Derraik J.G.B., Hofman P.L., Cutfield W.S. (2018). Antibiotics, gut microbiome and obesity. Clin. Endocrinol..

[15] James M.M., Pal N., Sharma P., Kumawat M., Shubham S., Verma V., Tiwari R.R., Singh B., Nagpal R., Sarma D.K. (2022). Role of butyrogenic Firmicutes in type-2 diabetes. J. Diabetes Metab. Disord..

[16] Bresser L.R.F., de Goffau M.C., Levin E., Nieuwdorp M. (2022). Gut Microbiota in Nutrition and Health with a Special Focus on Specific Bacterial Clusters. Cells.

[17] Alili R., Belda E., Fabre O., Pelloux V., Giordano N., Legrand R., Lassen P.B., Swartz T.D., Zucker J.D., Clément K. (2022). Characterization of the Gut Microbiota in Individuals with Overweight or Obesity during a Real-World Weight Loss Dietary Program: A Focus on the Bacteroides 2 Enterotype. Biomedicines.

[18] Cătoi A.F., Corina A., Katsiki N., Vodnar D.C., Andreicuț A.D., Stoian A.P., Rizzo M., Pérez-Martínez P. (2020). Gut microbiota and aging-A focus on centenarians. Biochim. Biophys. Acta (BBA)-Mol. Basis Dis..

[19] Gomes A.C., Hoffmann C., Mota J.F. (2020). Gut microbiota is associated with adiposity markers and probiotics may impact specific genera. Eur. J. Nutr..

[20] Nie K., Ma K., Luo W., Shen Z., Yang Z., Xiao M., Tong T., Yang Y., Wang X. (2021). Roseburia intestinalis: A Beneficial Gut Organism From the Discoveries in Genus and Species. Front. Cell. Infect. Microbiol..

[21] Hung W.C., Hung W.W., Tsai H.J., Chang C.C., Chiu Y.W., Hwang S.J., Kuo M.C., Chen S.C., Dai C.Y., Tsai Y.C. (2021). The association of targeted gut microbiota with body composition in type 2 diabetes mellitus. Int. J. Med. Sci..

[22] Lim S., Moon J.H., Shin C.M., Jeong D., Kim B. (2020). Effect of Lactobacillus sakei, a probiotic derived from kimchi, on body fat in koreans with obesity: A randomized controlled study. Endocrinol. Metab..

[23] Kim K.N., Yao Y., Ju S.Y. (2019). Short chain fatty acids and fecal microbiota abundance in humans with obesity: A systematic review and meta-analysis. Nutrients.

[24] Anachad O., Taouil A., Taha W., Bennis F., Chegdani F. (2023). The Implication of Short-Chain Fatty Acids in Obesity and Diabetes. Microbiol. Insights.

[25] Anggeraini A.S., Massi M.N., Hamid F., Ahmad A., As’ad S., Bukhari A. (2021). Effects of synbiotic supplement on body weight and fasting blood glucose levels in obesity: A randomized placebo-controlled trial. Ann. Med. Surg..

[26] Angelini G., Russo S., Mingrone G. (2024). Incretin hormones, obesity and gut microbiota. Peptides.

[27] Boer G.A., Holst J.J. (2020). Incretin Hormones and Type 2 Diabetes—Mechanistic Insights and Therapeutic Approaches. Biology.

[28] Cătoi A.F., Pârvu A.E., Mironiuc A., Silaghi H., Pop I.D., Andreicuț A.D. (2019). Ultra-early and early changes in bile acids and insulin after sleeve gastrectomy among obese patients. Medicina.

[29] Shemtov S.J., Emani R., Bielska O., Covarrubias A.J., Verdin E., Andersen J.K., Winer D.A. (2023). The intestinal immune system and gut barrier function in obesity and ageing. FEBS J..

[30] Cǎtoi A.F., Pârvu A.E., Andreicuț A.D., Mironiuc A., Crǎciun A., Cǎtoi C., Pop I.D. (2018). Metabolically healthy versus unhealthy morbidly obese: Chronic inflammation, nitro-oxidative stress, and insulin resistance. Nutrients.

[31] Rosendo-Silva D., Viana S., Carvalho E., Reis F., Matafome P. (2023). Are gut dysbiosis, barrier disruption, and endotoxemia related to adipose tissue dysfunction in metabolic disorders? Overview of the mechanisms involved. Intern. Emerg. Med..

[32] Vasques-Monteiro I.M.L., Silva-Veiga F.M., Miranda C.S., de Andrade Gonçalves É.C.B., Daleprane J.B., Souza-Mello V. (2021). A rise in Proteobacteria is an indicator of gut-liver axis-mediated nonalcoholic fatty liver disease in high-fructose-fed adult mice. Nutr. Res..

[33] Ju T., Bourrie B.C.T., Forgie A.J., Pepin D.M., Tollenaar S., Sergi C.M., Willing B.P. (2023). The Gut Commensal Escherichia coli Aggravates High-Fat-Diet-Induced Obesity and Insulin Resistance in Mice. Appl. Environ. Microbiol..

[34] Hartstra A.V., Schüppel V., Imangaliyev S., Schrantee A., Prodan A., Collard D., Levin E., Dallinga-Thie G., Ackermans M.T., Winkelmeijer M. (2020). Infusion of donor feces affects the gut–brain axis in humans with metabolic syndrome. Mol. Metab..

[35] Godsey T.J., Eden T., Emerson S.R. (2025). Ultra-Processed Foods and Metabolic Dysfunction: A Narrative Review of Dietary Processing, Behavioral Drivers and Chronic Disease Risk. Metabolites.

[36] Bevilacqua A., Speranza B., Racioppo A., Santillo A., Albenzio M., Derossi A., Caporizzi R., Francavilla M., Racca D., Flagella Z. (2025). Ultra-Processed Food and Gut Microbiota: Do Additives Affect Eubiosis? A Narrative Review. Nutrients.

[37] Monda A., Stefano M.I.D., Villano I., Allocca S., Casillo M., Messina A., Monda V., Moscatelli F., Dipace A., Limone P. (2024). Ultra-Processed Food Intake and Increased Risk of Obesity: A Narrative Review. Foods.

[38] Rondinella D., Raoul P.C., Valeriani E., Venturini I., Cintoni M., Severino A., Galli F.S., Mora V., Mele M.C., Cammarota G. (2025). The Detrimental Impact of Ultra-Processed Foods on the Human Gut Microbiome and Gut Barrier. Nutrients.

[39] Hall K.D., Ayuketah A., Brychta R., Walter P.J., Yang S., Zhou M., Hall K.D., Ayuketah A., Brychta R., Cai H. (2019). Ultra-Processed Diets Cause Excess Calorie Intake and Weight Gain: An Inpatient Randomized Controlled Trial of Ad Libitum Food Intake. Cell Metab..

[40] Konieczna J., Ruiz-Canela M., Galmes-Panades A.M., Abete I., Babio N., Fiol M. (2023). An Energy-Reduced Mediterranean Diet, Physical Activity, and Body Composition. Nutr. Obes. Exerc..

[41] Ichikawa T., Hashimoto Y., Igarashi Y., Kawai S., Kaji A., Sakai R. (2024). Association between gut microbiota and ultra-processed foods consumption among the patients with type 2 diabetes: A cross-sectional study. Nutr. Metab..

[42] Cuevas-Sierra A., Milagro I., Aranaz P., Alfredo J., Riezu-Boj J.I. (2021). Gut Microbiota Differences According to Ultra-Processed Food Consumption in a Spanish Population. Nutrients.

[43] Rauber F., Chang K., Vamos E.P., Laura M., Monteiro C.A., Millett C., Levy R.B. (2021). Ultra—processed food consumption and risk of obesity: A prospective cohort study of UK Biobank. Eur. J. Nutr..

[44] Köse S., Sözlü S., Bölükbaşi H., Ünsal N., Gezmen-Karadaǧ M. (2020). Obesity is associated with folate metabolism: A Systematic Review. Int. J. Vitam. Nutr. Res..

[45] Chan C.W., Chan P.H., Lin B.F. (2022). Folate Deficiency Increased Lipid Accumulation and Leptin Production of Adipocytes. Front. Nutr..

[46] Fu Y., Zhu Z., Huang Z., He R., Zhang Y., Li Y., Tan W., Rong S. (2023). Association between Vitamin B and Obesity in Middle-Aged and Older Chinese Adults. Nutrients.

[47] Rudzka A., Kapusniak K., Zielińska D., Kołożyn-Krajewska D., Kapusniak J., Barczyńska-Felusiak R. (2024). The Importance of Micronutrient Adequacy in Obesity and the Potential of Microbiota Interventions to Support It. Appl. Sci..

[48] Skrypnik K., Bogdański P., Sobieska M., Suliburska J. (2019). The effect of multistrain probiotic supplementation in two doses on iron metabolism in obese postmenopausal women: A randomized trial. Food Funct..

[49] Marks R. (2021). Vitamin C and obesity: Problems and solutions. Adv. Obes. Weight Manag. Control.

[50] Choi M.K., Song H.J., Paek Y.J., Lee H.J. (2013). Gender differences in the relationship between vitamin C and abdominal obesity: An observational study using Korea national health and nutrition examination survey, 2007–2010. Int. J. Vitam. Nutr. Res..

[51] Wilson R.B., Liang Y., Kaushal D., Carr A. (2024). Molecular Pharmacology of Vitamin C and Relevance to Health and Obesity—A Narrative Review. Int. J. Mol. Sci..

[52] Pereira S., Saboya C., Chaves G., Ramalho A. (2009). Class III obesity and its relationship with the nutritional status of vitamin A in pre- and postoperative gastric bypass. Obes. Surg..

[53] García O.P. (2012). Micronutrients, immunology and inflammation: Effect of vitamin A deficiency on the immune response in obesity. Proc. Nutr. Soc..

[54] Gomes C.D.C., Passos T.S., Morais A.H.A. (2021). Vitamin a status improvement in obesity: Findings and perspectives using encapsulation techniques. Nutrients.

[55] Aasheim E.T., Hofsø D., Hjelmesæth J., Birkeland K.I., Bøhmer T. (2008). Vitamin status in morbidly obese patients: A cross-sectional study. Am. J. Clin. Nutr..

[56] Botella-Carretero J.I., Alvarez-Blasco F., Villafruela J.J., Balsa J.A., Vázquez C., Escobar-Morreale H.F. (2007). Vitamin D deficiency is associated with the metabolic syndrome in morbid obesity. Clin. Nutr..

[57] Cătoi A.F., Iancu M., Pârvu A.E., Cecan A.D., Bidian C., Chera E.I., Pop I.D., Macri A.M. (2021). Relationship between 25 hydroxyvitamin d, overweight/obesity status, pro-inflammatory and oxidative stress markers in patients with type 2 diabetes: A simplified empirical path model. Nutrients.

[58] Vranić L., Mikolašević I., Milić S. (2019). Vitamin D deficiency: Consequence or cause of obesity?. Medicina.

[59] Taslim N.A., Graciela A.M., Harbuwono D.S., Syauki A.Y., Anthony A.N., Ashari N., Aman A.M., Tjandrawinata R.R., Hardinsyah H., Bukhari A. (2025). Zinc as a Modulator of miRNA Signaling in Obesity. Nutrients.

[60] Cui Z., Shao J., Zhu Z., Wang Y., Hu C., Yuan B. (2025). Nonlinear association of serum copper-zinc ratio with overweight/obesity in children and adolescents. Sci. Rep..

[61] Das T., Ahongshangbam R., Chabungbam R., Singh K.B. (2025). The Dual Edge of zinc: Linking excessive intake to obesity, diabetes, hypertension, and cardiovascular risks. Acta Biochim. Pol..

[62] Yang P., Yu S., Men L. (2025). Biochemical and Biophysical Research Communications Selenoproteins in adipose tissue and obesity. Biochem. Biophys. Res. Commun..

[63] Cheng L., Zeng Z., Quan H. (2025). Association of selenium with type 2 diabetes and obesity. Medicine.

[64] Zhang H., Dou B., Chen X., Sun X. (2025). Obesity, composite dietary antioxidant index, and their interactive association with the risk of cardiometabolic multimorbidity in the elderly from a large national survey. Lipids Health Dis..

[65] Mashayekhi Y., Jadhav A.N., Sarfraz M., Sachwani H., Khan M.A. (2025). Role of Serum Magnesium Deficiency in Insulin Resistance Among Overweight and Obese Children: A Meta-Analysis. Cureus.

[66] Piuri G., Zocchi M., Porta M.D., Ficara V., Manoni M., Zuccotti G.V., Pinotti L., Maier J.A., Cazzola R. (2021). Magnesium in Obesity, Metabolic Syndrome, and Type 2 Diabetes. Nutrients.

[67] Muralidharan J., Moreno-Indias I., Bulló M., Lopez J.V., Corella D., Castañer O., Vidal J., Atzeni A., Fernandez-García J.C., Torres-Collado L. (2021). Effect on gut microbiota of a 1-y lifestyle intervention with Mediterranean diet compared with energy-reduced Mediterranean diet and physical activity promotion: PREDIMED-Plus Study. Am. J. Clin. Nutr..

[68] Koutoukidis D.A., Jebb S.A., Zimmerman M., Otunla A., Henry J.A., Ferrey A., Schofield E., Kinton J., Aveyard P., Marchesi J.R. (2022). The association of weight loss with changes in the gut microbiota diversity, composition, and intestinal permeability: A systematic review and meta-analysis. Gut Microbes.

[69] Cani P.D., Everard A. (2016). Talking microbes: When gut bacteria interact with diet and host organs. Mol. Nutr. Food Res..

[70] Genton L., Cani P.D., Schrenzel J. (2015). Alterations of gut barrier and gut microbiota in food restriction, food deprivation and protein-energy wasting. Clin. Nutr..

[71] Kobylińska M., Antosik K., Decyk A., Kurowska K. (2022). Malnutrition in Obesity: Is It Possible?. Obes. Facts.

[72] Chapela S.P., Luciano A., Martinuzzi N., Llobera D., Ceriani F., Gonzalez V., Montalvan M., Verde L., Frias-Toral E., Pablo S. (2024). Obesity and micronutrients deficit, when and how to suplement. Food Agric. Immunol..

[73] Chao A.M., Quigley K.M., Wadden T.A. (2021). Dietary interventions for obesity: Clinical and mechanistic findings. J. Clin. Investig..

[74] Jalili M., Nazari M. (2023). Fermented Foods in the Management of Obesity: Mechanisms of Action and Future Challenges. Int. J. Med. Sci..

[75] Crovesy L., Ostrowski M., Ferreira D., Rosado E.L. (2017). Effect of Lactobacillus on body weight and body fat in overweight subjects: A systematic review of randomized controlled clinical trials. Int. J. Obes..

[76] Jung S., Lee K., Kang J., Yun S., Park H., Moon Y., Kim J. (2013). Effect of Lactobacillus gasseri BNR17 on Overweight and Obese Adults: A Randomized, Double-Blind Clinical Trial. Korean J. Fam. Med..

[77] Chu P.Y., Yu Y.C., Pan Y.C., Dai Y.H., Yang J.C., Huang K.C., Wu Y.C. (2024). The Efficacy of *Lactobacillus delbrueckii* ssp. bulgaricus Supplementation in Managing Body Weight and Blood Lipids of People with Overweight: A Randomized Pilot Trial. Metabolites.

[78] Ji Y., Chung Y.M., Park S., Jeong D., Kim B., Holzapfel W.H. (2019). Anti-obesity effects of Lactobacillus sakei in a diet induced obese murine model. PeerJ.

[79] Michael D.R., Davies T.S., Jack A.A., Masetti G., Marchesi J.R., Wang D. (2021). Daily supplementation with the Lab4P probiotic consortium induces significant weight loss in overweight adults. Sci. Rep..

[80] Mo S.J., Lee K., Hong H.J., Hong D.K., Jung S.H., Park S.D., Shim J.J., Lee J.L. (2022). Effects of Lactobacillus curvatus HY7601 and Lactobacillus plantarum KY1032 on Overweight and the Gut Microbiota in Humans: Randomized, Double-Blinded, Placebo-Controlled Clinical Trial. Nutrients.

[81] Effects T., Probiotics T., Hypertriglyceridemia D., Choi I., Kim S., Jeong J., Lee D.E., Huh C., Hong S.S., Sim J. (2016). Triglyceride-Lowering Effects of Two Probiotics, Lactobacillus plantarum KY1032 and Lactobacillus curvatus HY7601, in a Rat Model of High-Fat Diet-Induced Hypertriglyceridemia. J. Microbiol. Biotechnol..

[82] Zikou E., Dovrolis N., Dimosthenopoulos C., Gazouli M., Makrilakis K. (2023). The Effect of Probiotic Supplements on Metabolic Parameters of People with Type 2 Diabetes in Greece—A Randomized, Double-Blind, Placebo-Controlled Study. Nutrients.

[83] Ji Y., Park S., Chung Y., Kim B., Park H., Huang E., Jeong D., Jung H., Kim B., Hyun C. (2019). Amelioration of obesity-related biomarkers by Lactobacillus sakei CJLS03 in a high-fat diet-induced obese murine model. Sci. Rep..

[84] Wang M., Zhang B., Hu J., Nie S., Xiong T., Xie M. (2020). Intervention of five strains of Lactobacillus on obesity in mice induced by high-fat diet. J. Funct. Foods.

[85] Kai Y., Yung L., Lin H., Fu C., Tsung C., Yeh M., Shih W.L. (2022). *Lactobacillus delbrueckii* subsp. bulgaricus strain TCI904 reduces body weight gain, modulates immune response, improves metabolism and anxiety in high fat diet—induced obese mice. 3 Biotech.

[86] Narmaki E., Borazjani M., Ataie-Jafari A., Hariri N., Doost A.H., Qorbani M., Saidpour A. (2022). The combined effects of probiotics and restricted calorie diet on the anthropometric indices, eating behavior, and hormone levels of obese women with food addiction: A randomized clinical trial. Nutr. Neurosci..

[87] Solito A., Bozzi Cionci N., Calgaro M., Caputo M., Vannini L., Hasballa I., Archero F., Giglione E., Ricotti R., Walker G.E. (2021). Supplementation with Bifidobacterium breve BR03 and B632 strains improved insulin sensitivity in children and adolescents with obesity in a cross-over, randomized double-blind placebo-controlled trial. Clin. Nutr..

[88] Kondo S., Xiao J., Satoh T., Odamaki T., Takahashi S., Sugahara H., Yaeshima T., Iatsuki K., Kamei A., Abe K. (2010). Antiobesity Effects of Bifidobacterium breve Strain B-3 Supplementation in a Mouse Model with High-Fat Diet-Induced Obesity. Biosci. Biotechnol. Biochem..

[89] Vallianou N.G., Kounatidis D., Tsilingiris D., Panagopoulos F., Christodoulatos G.S., Evangelopoulos A., Karampela I., Dalamaga M. (2023). The Role of Next-Generation Probiotics in Obesity and Obesity-Associated Disorders: Current Knowledge and Future Perspectives. Int. J. Mol. Sci..

[90] Maioli T.U., Borras-Nogues E., Torres L., Barbosa S.C., Martins V.D., Langella P., Azevedo V.A., Chatel J.M. (2021). Possible Benefits of Faecalibacterium prausnitzii for Obesity-Associated Gut Disorders. Front. Pharmacol..

[91] Yang M., Wang J.H., Shin J.H., Lee D., Lee S.N., Seo J.G., Shin J.H., Nam Y.D., Kim H., Sun X. (2023). Pharmaceutical efficacy of novel human-origin Faecalibacterium prausnitzii strains on high-fat-diet-induced obesity and associated metabolic disorders in mice. Front. Endocrinol..

[92] Tajabadi-Ebrahimi M. (2017). Erratum: A Randomized Controlled Clinical Trial Investigating the Effect of Synbiotic Administration on Markers of Insulin Metabolism and Lipid Profiles in Overweight Type 2 Diabetic Patients with Coronary Heart Disease. Exp. Clin. Endocrinol. Diabetes.

[93] Moroti C., Francine L., Magri S., Costa M.D.R., Cavallini D.C.U. (2012). Effect of the consumption of a new symbiotic shake on glycemia and cholesterol levels in elderly people with type 2 diabetes mellitus. Lipids Health Dis..

[94] Kilic Yildirim G., Dinleyici M., Vandenplas Y., Dinleyici E.C. (2022). Effects of Multispecies Synbiotic Supplementation on Anthropometric Measurements, Glucose and Lipid Parameters in Children with Exogenous Obesity: A Randomized, Double Blind, Placebo-Controlled Clinical Trial (Probesity-2 Trial). Front. Nutr..

[95] Kießling G., Schneider J., Jahreis G. (2002). Long-term consumption of fermented dairy products over 6 months increases HDL cholesterol. Eur. J. Clin. Nutr..

[96] Taghizadeh M., Hashemi T. (2014). Synbiotic Food Consumption Reduces Levels of Triacylglycerols and VLDL, but not Cholesterol, LDL, or HDL in Plasma from Pregnant Women. Lipids.

[97] Rodriguez J., Hiel S., Neyrinck A.M., Le Roy T., Pötgens S.A., Leyrolle Q., Pachikian B.D., Gianfrancesco M.A., Cani P.D., Paquot N. (2020). Discovery of the gut microbial signature driving the efficacy of prebiotic intervention in obese patients. Gut.

[98] Sanchez M., Darimont C., Drapeau V., Emady-Azar S., Lepage M., Rezzonico E., Ngom-Bru C., Berger B., Philippe L., Ammon-Zuffrey C. (2014). Effect of Lactobacillus rhamnosus CGMCC1.3724 supplementation on weight loss and maintenance in obese men and women. Br. J. Nutr..

[99] Ranjha M.M.A.N., Shafique B., Batool M., Kowalczewski P.Ł., Shehzad Q., Usman M., Manzoor M.F., Zahra S.M., Yaqub S., Aadil R.M. (2021). Nutritional and health potential of probiotics: A review. Appl. Sci..

